# Partial Aminoglycoside Lesions in Vestibular Epithelia Reveal Broad Sensory Dysfunction Associated with Modest Hair Cell Loss and Afferent Calyx Retraction

**DOI:** 10.3389/fncel.2017.00331

**Published:** 2017-10-27

**Authors:** David R. Sultemeier, Larry F. Hoffman

**Affiliations:** ^1^Department of Head and Neck Surgery, David Geffen School of Medicine, University of California, Los Angeles, Los Angeles, CA, United States; ^2^Brain Research Institute, David Geffen School of Medicine, University of California, Los Angeles, Los Angeles, CA, United States

**Keywords:** ototoxicity, primary afferent, stimulus–response coherence, spontaneous discharge

## Abstract

Although the effects of aminoglycoside antibiotics on hair cells have been investigated for decades, their influences on the dendrites of primary afferent neurons have not been widely studied. This is undoubtedly due to the difficulty in disassociating pathology to dendritic processes from that resulting from loss of the presynaptic hair cell. This was overcome in the present investigation through development of a preparation using *Chinchilla laniger* that enabled direct perilymphatic infusion. Through this strategy we unmasked gentamicin’s potential effects on afferent calyces. The pathophysiology of the vestibular neuroepithelia after post-administration durations of 0.5 through 6 months was assessed using single-neuron electrophysiology, immunohistochemistry, and confocal microscopy. Hair cell densities within cristae central zones (0.5-, 1-, 2-, and 6-months) and utricle peri- and extrastriola (6-months) regions were determined, and damage to calretinin-immunoreactive calyces was quantified. Gentamicin-induced hair cell loss exhibited a profile that reflected elimination of a most-sensitive group by 0.5-months post-administration (18.2%), followed by loss of a second group (20.6%) over the subsequent 5.5 months. The total hair cell loss with this gentamicin dose (approximately 38.8%) was less than the estimated fraction of type I hair cells in the chinchilla’s crista central zone (approximately 60%), indicating that viable type I hair cells remained. Extensive lesions to afferent calyces were observed at 0.5-months, though stimulus-evoked modulation was intact at this post-administration time. Widespread compromise to calyx morphology and severe attenuation of stimulus-evoked afferent discharge modulation was found at 1 month post-administration, a condition that persisted in preparations examined through the 6-month post-administration interval. Spontaneous discharge was robust at all post-administration intervals. All calretinin-positive calyces had retracted at 2 and 6 months post-administration. We found no evidence of morphologic or physiologic recovery. These results indicate that gentamicin-induced partial lesions to vestibular epithelia include hair cell loss (ostensibly reflecting an *apoptotic effect*) that is far less extensive than the compromise to stimulus-evoked afferent discharge modulation and retraction of afferent calyces (reflecting *non-apoptotic effects*). Additionally, calyx retraction cannot be completely accounted for by loss of type I hair cells, supporting the possibility for direct action of gentamicin on the afferent dendrite.

## Introduction

The vestibulotoxic effects of aminoglycosides became apparent shortly after implementation of streptomycin therapy in the treatment of tuberculosis (Bignall et al., 1951; Robson and Goulding, 1952). Early investigations of aminoglycoside otopathology in animal models focused upon hair cells as the primary targets (Wersall and Hawkins, 1962; Lindeman, 1969a; Wersall et al., 1969; Watanuki et al., 1972). Since then, aminoglycosides have been widely used as research tools to lesion vestibular epithelia in investigations of the cellular and molecular mechanisms of hair cell regeneration (Weisleder and Rubel, 1992; Forge et al., 1993; Weisleder and Rubel, 1993; Lopez et al., 1999; Popper et al., 1999; Stone and Rubel, 2000; Dickman and Lim, 2004; Stone et al., 2004; Lyford-Pike et al., 2007; Haque et al., 2009; Kawamoto et al., 2009; Warchol, 2010b; Burns and Corwin, 2014; Warchol et al., 2017). Additionally, gentamicin is an agent commonly used in investigations of mammalian vestibular pathophysiology (Imamura and Adams, 2003a,b; Hirvonen et al., 2005; Hong et al., 2006; Day et al., 2007; Lue et al., 2009; Ding et al., 2010; Warchol, 2010a; Bremer et al., 2014; King et al., 2017). These studies have considerable translational value to enhance the understanding of gentamicin’s use in ablative therapy for intractable vertigo associated with Mèniére’s syndrome (Schuknecht, 1956, 1957; Nedzelski et al., 1993; Halmagyi et al., 1994; Minor, 1999; De Waele et al., 2002; Magnusson et al., 2007; Nguyen et al., 2009; Marques et al., 2015; Junet et al., 2016).

In most studies of aminoglycoside-induced vestibular pathophysiology, the lesions have been extensive with damage to hair cells, supporting cells, and afferent neurons. However, particular components of the mammalian vestibular neuroepithelia are believed to exhibit greater sensitivity to the deleterious effects of aminoglycosides than others. There is evidence suggesting that type I hair cells, particularly those in cristae central zones and utriclar striolae, are the most sensitive and are the first to be compromised or eliminated after aminoglycoside administration (Wersall and Hawkins, 1962; Lindeman, 1969b; Watanuki et al., 1972; Hirvonen et al., 2005). Lyford-Pike et al. (2007) provided evidence indicating that type I hair cells accumulated higher gentamicin concentrations than type II hair cells, supporting the notion that type I hair cells exhibit enhanced susceptibility to gentamicin toxicity than other constituents of the vestibular epithelia.

Although gentamicin toxicity to vestibular hair cells has been extensively investigated, few studies have focused upon the effects on Scarpa’s ganglion neurons and their dendrites within the sensory epithelia. Imamura and Adams (2003b) found only weak anti-gentamicin immunolabeling in Scarpa’s ganglion somata following both systemic or middle ear gentamicin administrations that produced strong immunolabeling in vestibular hair cells. Roehm et al. (2007) reported similar findings following gentamicin application to the round window in chinchillas (i.e., either by middle ear instillation or microcatheter delivery directed to the round window from an implanted osmotic pump). Harada et al. (1991) reported evidence of Scarpa’s ganglion degeneration after a 5-day course of middle ear gentamicin administration. In another investigation transtympanic gentamicin application led to increased levels of oxidative stress markers in the calyceal afferent endings (Hong et al., 2006). Hirvonen et al. (2005) used similar transtympanic applications of gentamicin in chinchillas to produce preparations in which mean hair cell density was reduced by 57% while virtually all afferent calyces were lost [see also Lyford-Pike et al. (2007)]. The remarkable finding from Hirvonen et al. (2005) was that afferent spontaneous discharge was preserved while evoked discharge was nearly eliminated, indicating that viable hair cells exhibited dramatic functional compromise not associated with hair cell apoptosis or necrosis (Li et al., 1995; Forge and Li, 2000; Matsui et al., 2002, 2003, 2004; Cunningham et al., 2004; Ding et al., 2010; Zhang et al., 2012; Tao and Segil, 2015).

Because the investigations by Hirvonen et al. (2005) and Lyford-Pike et al. (2007) utilized gentamicin doses that resulted in hair cell losses approaching 60% [i.e., approximating the proportion of type I hair cells (Desai et al., 2005a,b)], it could not be determined whether degeneration of afferent calyces stemmed from type I hair cell loss and subsequent postsynaptic degeneration, or whether it reflected direct and/or independent effects on calyces. Furthermore, neither of these studies tracked the fate of the afferent parent axons following gentamicin administration. Both issues are critical to the development of a more complete understanding of the cellular targets of aminoglycoside ototoxicity. That is, if afferent calyx loss is secondary to hair cell loss, and if the parent axons degenerate following calyx loss, then the cellular mechanisms of the lesions are likely to involve terminal cellular pathways in both hair cells and afferent neurons. However, if calyx damage exhibits some independence from hair cell loss, and if the parent axons remain within the epithelium, there must be intermediate levels of pathology that do not necessarily involve terminal (e.g., apoptotic or necrotic) mechanisms. These may be referred to as *non-apoptotic* effects. If the latter alternative is true, identifying epithelial constituents that are generally labile to other ototoxic agents, then there is hope for rehabilitation of vestibular hypofunction resulting from toxicity secondary to systemic aminoglycoside or other therapies. These issues were addressed in the present study through the development of a novel preparation enabling the use of refined gentamicin dosing that resulted in less extensive yet highly repeatable lesions than achieved in previous studies. The goal of these preparations was to use lower gentamicin doses to produce partial lesions enabling the distinction of hair cell and afferent pathology. Pathophysiologic correlates of these lesions were determined through single-afferent electrophysiology and immunohistochemical methodologies.

## Materials and Methods

### Experimental Animals, Surgical Preparation, and Gentamicin Administration

Adult male chinchillas (6–7 months of age, 0.4–0.6 kg body mass) were used for this study. These animals were acquired, cared for, and handled in accordance with the guidelines published in the NIH *Guide for the Care and Use of Laboratory Animals* (National Institutes of Health Publication revised 2011), and the principles presented in the *Guidelines for the Use of Animals in Neuroscience Research* by the Society for Neuroscience (available from the Society for Neuroscience). All procedures were approved by UCLA’s institutional animal care and use committee.

For the surgical implantation of a perilymph access port enabling direct gentamicin infusion, animals were anesthetized and placed on a platform equipped with a servo-controlled heater for core temperature maintenance (approximately 36.5°C) throughout the surgical preparation and gentamicin administration. Two anesthesia protocols were utilized during this study. For the early preparations, the protocol included administration of an intramuscular cocktail of ketamine and xylazine (30 and 4 mg/kg, respectively), followed by maintenance doses that amounted to 25% of the initial dose administered only as needed. For later preparations, isoflurane anesthesia (2–2.5%) was used exclusively. Once a surgical plane of anesthesia was achieved, the head was placed within a custom holder. A midline scalp incision was made to expose the surface of the tympanic bulla, and the bulla’s bony cap was removed to expose the middle ear. The chinchilla exhibits cavernous tympanic bullae with plenty of space between the prominent bony superior semicircular canal and the dorsal cap of the bulla. At the canal’s dorsal-most aspect, a small fenestra was carefully made into the perilymphatic space surrounding the membranous superior canal, into which a 5 mm length of 27-gauge stainless steel tubing was fit and secured with cyanoacrylate cement. The fenestra was made to provide patent access to the perilymphatic space surrounding the semicircular canal, but was not so large to allow the tubing to completely enter the superior semicircular canal and potentially occlude the duct. Once the cyanoacrylate cement cured, an epoxy-like bonding agent (Cerebond, 39465030; Leica Microsystems, Bannockburn, IL, United States) was poured around the cannula to secure it in place and fix the entire preparation to the surrounding temporal bone, leaving the top 1 mm of cannula exposed. By the time the bonding agent cured (approximately 5 min), perilymph was generally visualized at the top of the cannula. The cannula was fit with polyethylene tubing (PE-20) leading to a precision syringe placed in an infusion pump. A fixed volume of treatment solution (2.5 μl, composed of either 0.4 μg gentamicin/μl in Hank’s Balanced Saline Solution, HBSS, for lesioned specimens or HBSS alone for vehicle control specimens) was administered directly to the perilymphatic space over a 1-h period. Administration of 0.4 μg/μl gentamicin in 2.5 μl HBSS amounted to a total delivery of 1 μg gentamicin. The PE delivery tubing was then removed and the cannula sealed prior to replacing the bulla’s bony cap. The scalp incision was then sutured, whereupon the animal was removed from anesthesia and closely monitored until it regained sternal posture (<30 min). Upon full recovery from anesthesia, a minority of gentamicin treated specimens (*n* = 5/13) exhibited a slight head tilt toward the lesioned side within 2–3 postoperative days, whereas no vehicle control specimens (*n* = 3) exhibited head tilt. The head tilt resolved within 2 weeks after drug administration. Spontaneous nystagmus was not observed in any animal subjects.

### Vestibular Afferent Electrophysiology

The function of the vestibular epithelia in untreated, gentamicin-treated, or vehicle-control (i.e., HBSS only) preparations was assessed by electrophysiologic recording from individual afferent neurons following post-administration durations of 0.5, 1, 2, and 6 months. At the specified duration, animals were deeply anesthetized with a single intraperitoneal administration of sodium pentobarbital (50 mg/kg), which was sufficient to allow for cannulation of a jugular vein through which maintenance doses were administered as needed (0.05 cc, 50 mg/cc). A tracheotomy was performed for placement of an endotracheal tube into which a loosely fitting cannula delivering supplemental oxygen (100% O_2_) was placed. Core body temperature was maintained at 38°C via rectal probe and a custom servo-controlled heating system. Each animal’s head was held in a stereotyped position (10° right ear down, 15° nose down) by a custom holder fixed to a servo-controlled rotation table. This position was maintained for each experimental preparation. Heart and respiratory rates, as well as blood oxygen saturation, were recorded at regular intervals throughout the recording session.

Recordings from individual vestibular afferent neurons were made by exposing the right superior vestibular nerve in the region of Scarpa’s ganglion through a fenestra made in the internal vestibular meatus, approximately 1mm medial to the superior and horizontal semicircular canal ampullae. Vestibular afferent discharge was recorded using glass microelectrodes pulled to impedances of approximately 40 MΩ when filled with 1 M KCl. With the animal restrained in the custom head holder, afferent discharge was recorded in the absence of head movement stimuli (i.e., spontaneous discharge) and during a broad repertoire of stimulus rotations. Afferents projecting from the horizontal and superior cristae and from the utricle could be recorded from this site in the superior vestibular nerve, which were easily distinguished in normal animals based upon their discharge modulation during manual turntable rotations. That is, in untreated preparations horizontal canal afferents were identified by increased discharge rate during turntable rotations producing utriculopetal relative endolymph flow, while superior canal afferents exhibited decreased discharge rate with similar rotations (this phase relationship is illustrated in **Figure 2**). Utricular afferents were identified as those that were unresponsive to turntable rotations, or responded at twice the stimulus frequency (i.e., increased or decreased discharge with rotations in either direction).

Spontaneous and stimulus-evoked spike trains were first analyzed using methods routine for studies of vestibular afferents neurons. For spontaneous discharge, interspike intervals (ISIs) were calculated during a 20 s recording epoch, from which the mean and standard deviation were determined. The coefficient of variation (CV) for each afferent was then computed as the ratio of ISI standard deviation to ISI mean. The perstimulus discharge spike trains were first analyzed using discrete Fourier analysis. However, upon finding that the responses in gentamicin-treated specimens were severely attenuated or non-existent, it became clear that methods were needed to objectively verify a statistical correlation between stimulus and response. To achieve greater measure of confidence in evaluating very weak responses, we adapted the frequency-domain measures for determining stimulus–response coherence computed directly from the spike trains (Jarvis and Mitra, 2001).

As in all experiments employing glass microelectrodes and axonal recordings, an inherent sampling bias exists toward the larger axons in the chinchilla’s superior vestibular nerve. Despite this potential bias, our recordings from untreated specimens suggest that it is offset with methods (e.g., use of high-impedance electrodes) resulting in a proclivity for afferents with low values of CV, indicative of smaller caliber afferents (Baird et al., 1988). The inherent bias toward sampling afferents with larger axons, together with the implementation of methods with demonstrable capabilities for recording from smaller afferents, result in a paradigm designed for sampling the broad distribution of axon diameters that are found in the chinchilla’s vestibular nerve (Hoffman and Honrubia, 2002).

### Histology and Whole-Mount Immunohistochemistry

We utilized fluorescence immunohistochemistry and confocal microscopy to visualize the cytoarchitecture of chinchilla vestibular sensory epithelia. Vestibular endorgans were prepared as whole mounts and incubated with an antibody to calretinin (anti-CALB2) to immunolabel calyx-only afferents, and, in some specimens, an antibody to β3-tubulin (anti-TUBB3) to label all calyces and nerve fibers. In contrast to murine vestibular organs in which most hair cells are calretinin-immunopositive (CALB2+) (Desai et al., 2005a,b), only a subset of afferent calyces (i.e., those associated with calyx-only afferents) are CALB2+ in chinchilla vestibular epithelia (Desmadryl and Dechesne, 1992). We used CALB2 immunohistochemistry to delimit cristae central zones and utricular striola regions, and quantified gentamicin-induced modifications to hair cells and afferents in these regions which reportedly were most sensitive to gentamicin (Chen et al., 1999; Hilton et al., 2002).

After specified post-administration durations and electrophysiologic recording sessions, animals were euthanized (sodium pentobarbital overdose) and temporal bones were quickly dissected. Temporal bones were immediately infused with 4% paraformaldehyde (in 0.1 M phosphate buffer) through the oval window, after which the roof of the vestibule (floor of the subarcuate fossa) was removed prior to incubating in fixative overnight at 4°C. After three washes in 0.1 M phosphate buffered saline (PBS; pH 7.4), vestibular epithelia and nerve branches were microdissected. The tissues were incubated in blocking solution (0.25% Triton-X100, 1.0% BSA solution in PBS) for 2 h at room temperature, and then incubated 48–72 h at 4°C in a primary antibody cocktail that included combinations of the following antibodies diluted in blocking solution: 1:250 mouse anti-CALB2 (MAB1568; Millipore, Billerica, MA, United States) or 1:250 rabbit anti-CALB2 (AB5054; Millipore), 1:250 rabbit anti-Class III β-TUBB3 (PRB-435P; Covance, Princeton, NJ, United States), 1:250 rabbit anti-Myosin VI (MYOVI; 25-6791, Proteus Biosciences, Ramona, CA, United States). After washing in PBS, specimens were incubated for 2 h at room temperature in combinations of the following secondary antibodies and stains diluted in blocking solution: 1:500 Alexa Fluor 633 conjugated goat anti-mouse antibody (A-21050; Invitrogen, Carlsbad, CA, United States), 1:500 Alexa Fluor 546 goat anti-rabbit antibody (A-11010; Invitrogen), 1:150 NeuroTrace 500/525 green fluorescent Nissl stain (N-21480; Invitrogen). Tissue was mounted on glass slides with 1–2 spacers (S-24735; Invitrogen) in Vectashield Hard Set Mounting Medium (H-1500; Vector Laboratories, Burlingame, CA, United States).

#### Antibody Specificity

Secondary-only controls for all antibodies were processed alongside regular staining to ascertain background fluorescence. No immunolabeling was observed in any tissues processed without the addition of primary antibody (not shown).

The two anti-CALB2 antibodies utilized in the present study were generated against recombinant rat Calretinin (manufacturer’s technical information). We confirmed antigen specificity of the antibodies by staining cryosections of mouse cerebellum (data not shown). In these controls we observed immunolabeling consistent with previous investigations (Miyata et al., 1999; Bearzatto et al., 2006), whereby anti-CALB2 antibody positively labeled granule cells but not Purkinje cells, the latter of which are positive for calbindin (a very similar but distinct calcium-binding protein). Furthermore, the staining pattern that we observed in the control chinchilla vestibular epithelia was consistent with that observed in previous studies (Desmadryl and Dechesne, 1992; Desai et al., 2005a,b).

The anti-TUBB3 antibody was produced against microtubules isolated from rat brain (manufacturer’s technical information). This antibody has been widely used as a neuron specific marker and does not bind β-tubulin found in glia (manufacturer’s technical information). Western blot analyses of the antibody against β3-tubulin demonstrated that this antibody recognized a doublet of bands in spiral ganglion extracts (Flores-Otero et al., 2007) consistent with a post-translational modification of β3-tubulin detected by this antibody (Cicchillitti et al., 2008). As previously reported, we did not observe staining of non-neuronal cells (i.e., support cells or hair cells).

The anti-MYOVI antibody was generated against amino acids 1049–1254 of porcine MYOVI (manufacturer’s technical information). Anti-MYOVI is commonly used as a marker for hair cells including vestibular types I and II (Hasson et al., 1997). Consistent with this and other studies, we observed specific hair cell labeling with this antibody in chinchilla vestibular epithelia.

### Confocal Microscopy and Analysis

Confocal images were captured on a Zeiss LSM 510 Meta confocal microscope implemented on an upright Axioplan 2 microscope using Zeiss LSM 510 software. The 488 nm (15% intensity), 543 nm (80–100% intensity) and 633 nm (10–20% intensity) laser lines were used for excitation. Bandpass filters of 505–530 nm, 560–615 nm, and a longpass filter 650 nm were used to capture separate emission channels. A Zeiss Plan-Neofluar 10X/0.3 NA objective was used to capture low-magnification images and high magnification images were obtained using a Zeiss Plan-Apochromat 63X/1.4 NA oil-immersion objective. Confocal stack micrographs were prepared for publication using Volocity software (Perkin Elmer, Waltham, MA, United States). Adobe Photoshop 7.0.1 (Adobe Systems Incorporated, San Jose, CA, United States) was used to compile figures.

#### Morphometric Analyses

Parent axons and calyces of CALB2+ afferents were counted using the neuron tracing application in *Neurolucida* (MBF Bioscience; Williston, VT, United States). Hair cell quantification and sensory epithelia area measurements were completed using automated and semi-automated methods implemented within the *Volocity* software environment (PerkinElmer/Improvision, Waltham, MA, United States). Hair cell nuclei were differentiated from support cell nuclei based on position within the sensory epithelium, morphology, and chromatin condensation (i.e., manifested in Nissl stain fluorescence intensity). Support cell nuclei were cuboidal, had compact chromatin, and formed a tightly packed monolayer over the basement membrane extending to the transitional epithelia at the ends of the sensory epithelia. In contrast, nuclei of hair cells were usually spherical, have less compact chromatin, and were located more toward the lumen compared to support cell nuclei.

Hair cell nuclei within crista central zones and utricular peri- and extrastriola regions were included in density measurements. Cristae central zones were approximated as the area of sensory epithelia innervated by CALB2+ afferents. Two – four confocal stacks were sampled from each crista. Four utricle peristriola regions were systematically evaluated along the rostral to caudal line of polarity reversal (LPR), and encompassed the striola, defined as the region harboring CALB2+ calyces, and immediately adjacent areas. Two confocal stacks each from the lateral and medial striola were used to estimate hair cell densities in these regions. The borders of these image stacks constituted the counting frames for quantification. Nuclei contacting either of two adjacent sides of the counting frames were excluded from quantification, while nuclei contacting the other two sides were included in the counts. This estimation procedure was implemented to compensate for incomplete counting units transected by the counting frame. Support cell densities were quantified using similar methods to evaluate the treatment-induced alterations of the epithelia surface areas.

We found that intraperilymphatic gentamicin administration resulted morphologic distortion in afferent calyces, and therefore established criteria on which to identify an “intact” calyx for quantification purposes. A CALB2+ calyx was counted if its height (i.e., along the base-neck hair cell axis) extended to or above the apical-most level of the Nissl-stained hair cell nucleus. Density measures were computed as fractions of contralateral control density, and are also presented as CALB2+ calyces or axons per 100 μm^2^ of epithelium.

The areas of sensory epithelia used to determine densities of hair cells and CALB2+ parent axons and calyces were established by measuring the width along the support cell nuclei layer every 24 μm across the length of the cristae central zone (estimated by presence of CALB2+ parent axons) and utricle. The area was estimated as the product of the average width and the length.

#### Correlates between Afferent Electrophysiology and Vestibular Epithelia Morphology

It should be clarified that the characteristics of afferent electrophysiology and sensory epithelia morphology are intended to be representative of each metric within a given specimen. For most specimens, stimulus-evoked discharge modulation was lost or severely attenuated, and in all but a limited number of records (e.g., **Figures 3C, 4A-v,vi**) it was not possible to distinguish the epithelial origin of a given afferent. Furthermore, morphologic analyses were restricted to the central zones of the cristae. Direct associations between afferent discharge and morphologic characteristics of these crista regions were not made.

### Retrograde Labeling of Vestibular Afferent Dendrites

To confirm the presence of putative boutons within crista central zones and utricular striolae, representing the intact components of dimorphic afferents, we performed extracellular injections of 2% TRITC-conjugated biotin (T12092, Molecular Probes Inc.) in two gentamicin-dosed specimens. This was accomplished through pneumatic injection of the label directly into the superior vestibular nerve in the vicinity of Scarpa’s ganglion (i.e., approximately 2 mm medial to the superior and horizontal ampullae). The label was allowed to incubate for 6 h, after which normal fixation and tissue harvesting procedures were completed.

### Statistical Analyses

#### Analyses and Comparisons of Afferent Discharge Characteristics

In this report most of the electrophysiologic data are represented as epochs of instantaneous discharge rates under the different gentamicin exposure conditions. The goal of these illustrations was to demonstrate the perstimulus discharge associated with the morphologic analyses. All instantaneous discharge rate waveforms were based upon spiketimes and derived from a Gaussian local rate filter using a 6.4 Hz corner frequency (Paulin and Hoffman, 2001). Mean spontaneous discharge rates (e.g., **Figure 4**) were computed as the reciprocal of ISI mean. Most perstimulus discharge records were obtained during 0.8 Hz, with the exception of **Figure 4B** for which the records were obtained during 0.4 Hz rotations.

Hirvonen et al. (2005) demonstrated that gentamicin eliminated the responses of vestibular afferents to head movement stimuli, while spontaneous discharge was preserved. As indicated above, our recording paradigm was conducted in head-fixed preparations on a turntable that precluded the ability to identify utricular afferents through the modulation of linear acceleration stimuli. In untreated preparations afferents projecting from the utricle were most often identified as those unresponsive to rotational stimuli. Therefore, the challenge in the present study was interpreting the absence of rotational responses as those resulting from the effects of gentamicin from a utricular afferent in our recording paradigm. We addressed this challenge by: (1) providing a perspective of the relatively low probability of recording a utricular afferent at the recording site; and (2) through analyses of previously published data of semicircular canal and utricular afferents (Baird et al., 1988; Goldberg et al., 1990).

For analyses of afferent discharge from gentamicin-treated vestibular epithelia it was important to have samples of untreated (normal) afferents that projected within the chinchilla’s superior vestibular nerve accessible at the recording site. This included afferents from the horizontal and superior cristae and the utricle. Data for untreated cristae afferents came from our laboratory’s database that included 431 neurons projecting from horizontal and superior cristae. In addition, the spontaneous discharge characteristics of canal and utricular afferents were obtained from earlier investigations (Baird et al., 1988; Goldberg et al., 1990). Using image analysis software (ImageJ 1.38g), we reconstituted CV and mean ISI values of 438 semicircular canal afferents (Baird et al., 1988), and 342 utricular afferents (Goldberg et al., 1990). The reconstituted values were compared to reported summaries of the original data. For example, the mean discharge rate of 251 *regular* afferents of the original utricular afferent dataset (i.e., those with CV^∗^ < 0.1) was reported to be 54.2 ± 1.0 spikes⋅s^-1^ [standard error as originally reported; (Goldberg et al., 1990)], and the reconstituted dataset of 238 regular afferents exhibited a similar mean discharge rate (±standard error) of 54.6 ± 1.07 spikes⋅s^-1^. These analyses indicated that reconstitution of the published datasets produced a representative facsimile of the published data.

As specified above, coherence analyses were used to statistically verify stimulus-evoked discharge modulation. These measures were based upon multitaper spectral analyses (Jarvis and Mitra, 2001), and were primarily used to determine whether the perstimulus discharge of afferents in gentamicin-treated specimens represented statistically verifiable responses to discrete sine rotational stimuli. Coherence measures of the discharge from untreated afferents were also conducted to illustrate the sensitivity and veracity of this analytical strategy in identifying stimulus-evoked responses from challenging perstimulus discharge data (e.g., those exhibiting low sensitivity measures during low stimulus magnitudes).

The distributions of spontaneous discharge characteristics were compared by computing Kullback–Leibler divergences (KLDs). KLD is a non-symmetrical measure of the difference between two probability distributions. For two discrete probability distributions *P* and *Q*, KLD is computed from the formula (Mackay, 2003):

$$\begin{array}{c} \mathrm{KLD}(P||Q)=\sum_{i}P_{i}\log\frac{P_{i}}{Q_{i}} \end{array}$$

Therefore, in the present study we established the convention that distributions representing semicircular canal afferents conformed to *P*, while the compared distributions conformed to *Q*. A resampling strategy was implemented to test hypotheses that the KLD between two original distributions could have been derived from random sampling from the combined distributions (i.e., no difference between the original distributions). Software scripts used to compute KLD and manage the bootstrap resampling were written in the *Igor* environment (Wavemetrics Inc., Lake Oswego, OR, United States). The resampling strategy was based upon one million resamples and subsequent KLD calculations. Probabilities to support the null hypothesis (specified above) were determined explicitly, except when the KLDs from the original distributions were outside the range of the distribution of KLDs generated from one million random resamples. In these cases, the probability is expressed as an inequality (i.e., *p* < 10^6^). Values less than 0.05 support rejection of the null hypothesis stated above, indicating that the two distributions could not be derived by random sampling from a dataset constituted from the combined distributions (i.e., they were different).

#### Comparisons of Morphometric Parameters

Statistical comparisons of the different treatment groups were done by analysis of variance (single and multifactor ANOVA) with a Newman–Keuls *post hoc* test and performed using GraphPad Prism 6.0 software (GraphPad Software, San Diego, CA, United States). Data are presented as mean ± standard deviation (SD). Observed significance levels (*p-*value) are indicated in the figures with asterisks coded as follows: single asterisk (^∗^) reflects *p* < 0.05, double asterisks (^∗∗^) reflect *p* < 0.01, and triple asterisks (^∗∗∗^) reflect *p* < 0.001.

## Results

### Morphology and Physiology of Non-lesioned Crista Epithelia and Afferent Dendrites

#### Cytoarchitecture of the Chinchilla Crista Central Zones

**FIGURE 1:**
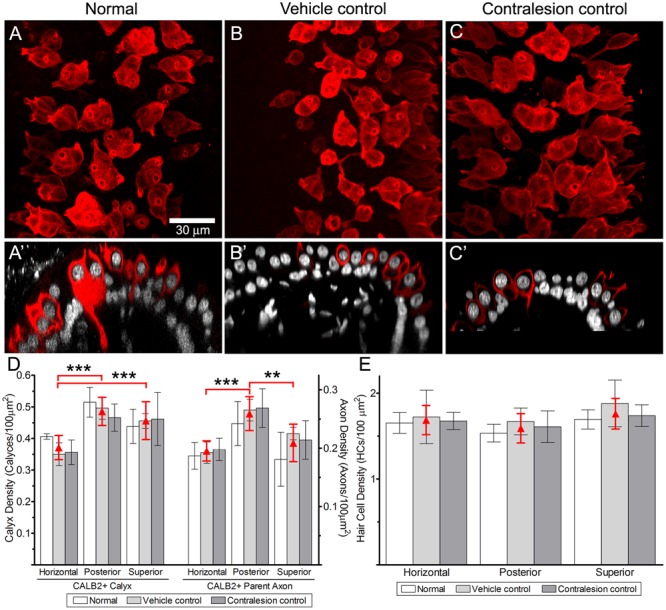
Comparison of CALB2+ calyces within cristae central zones from Normal, Vehicle Control, and Contralesion control specimens. The micrographs represent maximum intensity projections **(A–C)** and orthogonal optical sections **(A′–C′)** of whole-mounted horizontal semicircular canal cristae labeled with anti-CALB2 (red) and Nissl stain (grayscale). Calyx morphology was similar in all control specimens, and exhibited narrow fluted necks at their apex closely conforming to the type I hair cell morphology. Scale bar shown in **(A)** applies to all micrographs. **(D)** CALB2+ calyx and parent axon densities expressed as counts/100 μm^2^ epithelial area for horizontal, posterior, and superior cristae in all control specimens. See text for a discussion of the differences across epithelia. CALB2+ calyx densities were similar across all control conditions. **(E)** Central zone hair cell densities were similar across crista type and control conditions. The three control conditions were similar within any epithelium for each measure, and therefore the red triangles (and error bars) represent the mean control densities (±*SD*) over all conditions.

The cytoarchitecture of central zone epithelia from non-lesioned chinchilla cristae are presented first to provide the histologic perspective for the balance of the physiologic and morphologic correlates presented in this investigation. This was achieved through quantitative morphometry of normal (untreated), vehicle control (HBSS-infused) and contralesion control (epithelia contralateral to gentamicin infusion) cristae. In addition, these data provided the basis to determine whether the intraperilymphatic administration procedure induced histologic or physiologic changes that could have influenced our analyses independent of gentamicin’s effects. Maximum intensity projections of confocal image stacks from the central zones of CALB2-immunolabeled horizontal cristae, representing the three control groups, are shown in **Figures 1A–C**. In each control condition similar simple and complex calyx arbor morphologies (Fernandez et al., 1988) were observed. Also shown are corresponding orthogonal optical sections through each confocal stack (**Figures 1A′–C′**) to further illustrate similarities in CALB2+ calyx morphology among these control groups. These micrographs demonstrate that CALB2+ calyces in non-lesioned epithelia were strongly labeled from the parent axons to the fluted calyceal necks, which extended well above the nuclei of encapsulated hair cells.

Quantitative estimates of the distributions of CALB2+ calyces, CALB2+ parent axons, and hair cells were expressed as densities (count per 100 μm^2^ area of the crista central zone), and compared (two-factor repeated measures ANOVA) across control condition (i.e., untreated normal, vehicle control, and contralesion control) and crista identity (i.e., horizontal, posterior, and superior). We found that the densities of each epithelial component were similar across control conditions as shown in **Figures 1D,E** and **Tables 1, 2** (*p* > 0.5). The similarities between vehicle controls and normal epithelia indicate that the chronic placement of the perilymph access port and HBSS infusion did not result in alterations of the morphologic parameters analyzed. Furthermore, despite the presence of a patent vestibular aqueduct in the chinchilla (Roehm et al., 2007) the similarities between contralesion controls and normal epithelia indicated that administration of HBSS, and low gentamicin doses as compared later, did not alter these morphologic indices in the contralateral epithelia. This supports the use of contralesion control specimens for direct comparison of the lesions induced by the intraperilymphatic gentamicin administration.

**Table 1:** CALB2+ parent axon and calyx densities.

Treatment	Density (units/100 μm^2^)					
	CALB2+ calyx			CALB2+ parent axon		
	Horizontal	Posterior	Superior	Horizontal	Posterior	Superior
Normal	0.41 ± 0.009 (*N* = 3)	0.47 ± 0.027 (*N* = 3)	0.44 ± 0.054 (*N* = 3)	0.19 ± 0.023 (*N* = 3)	0.24 ± 0.038 (*N* = 3)	0.18 ± 0.046 (*N* = 3)
Vehicle control	0.35 ± 0.036 (*N* = 3)	0.50 ± 0.035 (*N* = 3)	0.46 ± 0.023 (*N* = 3)	0.19 ± 0.018 (*N* = 3)	0.27 ± 0.019 (*N* = 3)	0.23 ± 0.011 (*N* = 3)
Contralesion control	0.36 ± 0.039 (*N* = 5)	0.47 ± 0.043 (*N* = 8)	0.46 ± 0.084 (*N* = 5)	0.20 ± 0.019 (*N* = 5)	0.27 ± 0.033 (*N* = 8)	0.21 ± 0.033 (*N* = 5)
Combined control	0.37 ± 0.039 (*N* = 11)	0.48 ± 0.045 (*N* = 14)	0.45 ± 0.060 (*N* = 11)	0.19 ± 0.019 (*N* = 11)	0.26 ± 0.031 (*N* = 14)	0.21 ± 0.035 (*N* = 11)
0.5 month	0.12 ± 0.210^∗^ (*N* = 3)	0.19 ± 0.320^∗∗∗^ (*N* = 3)	0.18 ± 0.311^∗∗^ (*N* = 3)	0.20 ± 0.032 (*N* = 3)	0.26 ± 0.014 (*N* = 3)	0.21 ± 0.021 (*N* = 3)
1 month	0.11 ± 0.186 (*N* = 3)	0.11 ± 0.172^∗∗^ (*N* = 3)	0.12 ± 0.200^∗^ (*N* = 3)	0.17 ± 0.044 (*N* = 3)	0.23 ± 0.031 (*N* = 3)	0.20 ± 0.028 (*N* = 3)
2 months	0.00 ± 0.000^∗∗∗^ (*N* = 3)	0.00 ± 0.000^∗∗∗^ (*N* = 3)	0.00 ± 0.000^∗∗∗^ (*N* = 2)	0.15 ± 0.009 (*N* = 3)	0.22 ± 0.035 (*N* = 3)	0.20 ± 0.037 (*N* = 2)
6 months	0.00 ± 0.000^∗∗∗^ (*N* = 3)	0.00 ± 0.000^∗∗∗^ (*N* = 4)	0.01 ± 0.015^∗∗∗^ (*N* = 4)	0.19 ± 0.019 (*N* = 3)	0.23 ± 0.027 (*N* = 4)	0.17 ± 0.054 (*N* = 4)

*All values are means ± SD. *N*, number of specimens. Significance values represent comparisons with combined controls. These data complement the relative densities depicted in **Figure 8**. Asterisks correspond to: ^∗^*p* < 0.05; ^∗∗^*p* < 0.01; ^∗∗∗^*p* < 0.001.*

**Table 2:** Hair cell densities in the cristae central zones.

Treatment	Hair cell density (hair cell nuclei/100 μm^2^)			
	Horizontal	Posterior	Superior	Average
Normal	1.65 ± 0.122 (*N* = 3)	1.53 ± 0.102 (*N* = 3)	1.69 ± 0.112 (*N* = 3)	1.62 ± 0.071 (*N*^∗^ = 3)
Vehicle control	1.72 ± 0.311 (*N* = 3)	1.67 ± 0.156 (*N* = 2)	1.88 ± 0.272 (*N* = 3)	1.79 ± 0.153 (*N*^∗^ = 3)
Contralesion control	1.67 ± 0.100 (*N* = 5)	1.61 ± 0.184 (*N* = 8)	1.74 ± 0.130 (*N* = 5)	1.69 ± 0.066 (*N*^∗^ = 5)
Combined control	1.68 ± 0.165 (*N* = 11)	1.60 ± 0.160 (*N* = 13)	1.77 ± 0.172 (*N* = 11)	1.70 ± 0.107 (*N*^∗^ = 11)
0.5 month	1.43 ± 0.167 (*N* = 3)	1.40 ± 0.226 (*N* = 3)	1.32 ± 0.321 (*N* = 3)	1.39 ± 0.231^∗∗^ (*N* = 3)
1 month	1.34 ± 0.388 (*N* = 3)	1.24 ± 0.146 (*N* = 3)	1.18 ± 0.244 (*N* = 3)	1.24 ± 0.276^∗∗^ (*N* = 3)
2 months	1.14 ± 0.066 (*N* = 3)	1.24 ± 0.121 (*N* = 3)	1.17 ± 0.035 (*N* = 2)	1.17 ± 0.032^∗∗∗^ (*N* = 3)
6 months	1.03 ± 0.255 (*N* = 3)	1.06 ± 0.208 (*N* = 4)	1.05 ± 0.113 (*N* = 3)	1.04 ± 0.171^∗∗∗^ (*N* = 4)

*All values are means ± SD. *N*, number of specimens; *N*^∗^, number of specimens with >2 cristae counted. Average represents hair cell density calculated when cristae from a specimen were treated as a single epithelium. Significance values represent comparisons with combined controls. These data complement that depicted in **Figure 9**. Asterisks correspond to: ^∗∗^*p* < 0.01; ^∗∗∗^*p* < 0.001.*

Though not a principal objective of this investigation, our analyses revealed differences in CALB2+ calyx and parent axon densities across cristae type (**Figure 1D** and **Table 1**). While CALB2+ calyx densities (calyces/100 μm^2^) were similar in the posterior (0.48 ± 0.045; *N* = 14) and superior (0.45 ± 0.06; *N* = 11) cristae, mean CALB2+ calyx density of the horizontal cristae (0.37 ± 0.039; *N* = 11) was approximately 20% less than the vertical cristae (*p* < 0.001). Additionally, CALB2+ axon density (axons/100 μm^2^) was approximately 25% higher in posterior canal cristae (0.26 ± 0.031; *N* = 14) than in horizontal (0.19 ± 0.019, *N* = 11; *p* < 0.001) and superior (0.21 ± 0.035, *N* = 11; *p* < 0.01) cristae. In contrast to these differences in CALB2+ afferent innervation, mean central zone hair cell densities (HCs/100 μm^2^) were similar across all control cristae (*p* > 0.05; **Figure 1E** and **Table 2**). These data suggest that the chinchilla superior cristae receive putative calyx-only afferents with a larger number of complex calyces compared to horizontal cristae. Additionally, the increased number of CALB2+ calyces in the posterior cristae, accompanied by an increase in parent axon density, reflects a greater number of putative calyx-only afferents.

#### Afferent Electrophysiology in Non-lesioned Specimens

**FIGURE 2:**
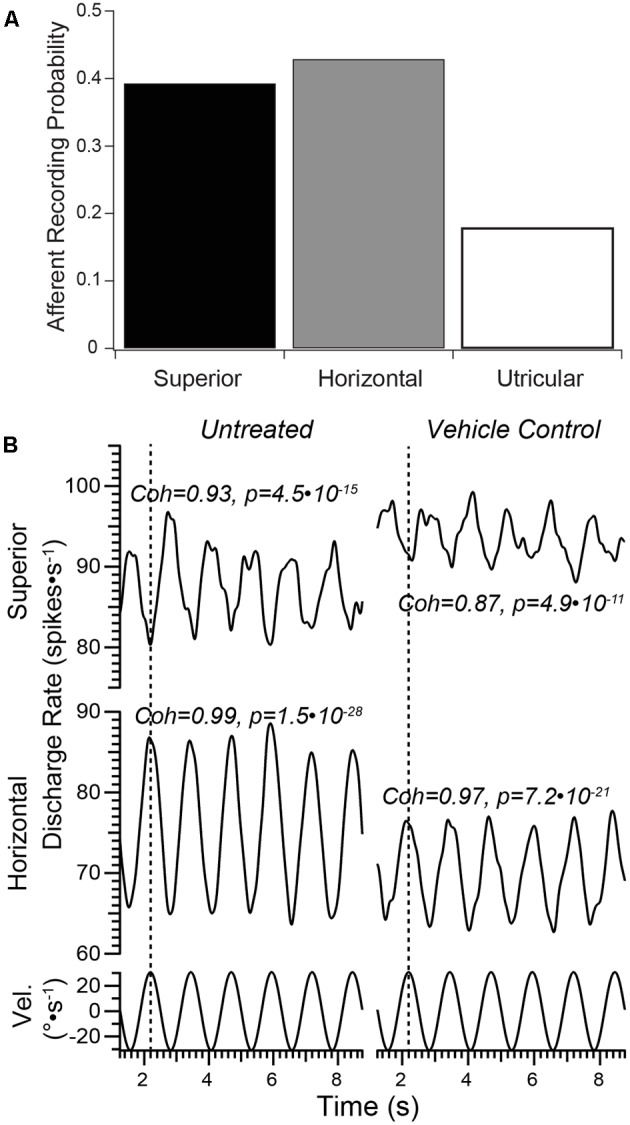
Vestibular afferent responses in untreated and vehicle controls. **(A)** The probability of encountering an afferent neuron projecting from each of the three epithelia served by the superior vestibular nerve in normal, untreated preparations is shown in this bar graph. This provided a perspective for the afferents probed at the recording site in untreated specimens that would be unresponsive to rotational stimuli (e.g., putative utricular afferents). These data showed that the majority of afferents accessible to microelectrode recording at this site projected from either the superior or horizontal cristae, which were readily identified by turntable rotation. Less than 20% of neurons encountered were unresponsive to rotation, which were identified as projecting from the utricle. **(B)** Afferents recorded from *Vehicle Control* specimens exhibited stimulus-evoked modulation similar to *Normal* (untreated) controls. Representative afferents that projected from the superior and horizontal cristae in one preparation are shown demonstrating robust modulation in response to sinusoidal turntable rotation (sinusoidal angular velocity stimulus trajectories, in °s^-1^, represented in the bottom traces). The scale of the vertical axes are similar in absolute range. Note the responses of the superior and horizontal afferents were approximately 180° out of phase (denoted by the dashed vertical line along each horizontal axis), illustrating the distinguishing characteristic of afferents projecting from these neuroepithelia. Afferents projecting from each crista in *Vehicle Control* specimens exhibited similarly robust modulation. Coherence and associated *p*-values for each full record are shown.

The electrophysiology of non-lesioned chinchilla vestibular afferent neurons provides the background for evaluating the alterations concomitant with low-dose gentamicin administration. This background is provided primarily by our laboratory’s database of afferents recorded in normal, untreated specimens (**Figures 2A,B** *Untreated*). In addition, a population of non-lesioned afferents were derived from the vehicle-control specimens described above. These serve to test whether the surgical implantation of the perilymph port into the bony superior semicircular canal or the infusion of HBSS resulted in deleterious effects upon the physiology of superior and horizontal semicircular canal afferents. As discussed above, no differences were found in the morphology of CALB2+ calyces and hair cell densities in these specimens.

Our experience in recording from over 400 semicircular canal afferents in normal, untreated specimens also served as the basis for establishing the probability of encountering afferents at the recording site within the superior vestibular nerve that projected to specific epithelia (i.e., the superior and horizontal cristae and the utricle, **Figure 2A**). In untreated specimens, the recording procedures utilized herein revealed afferent discharge characteristics similar to those previously reported (Baird et al., 1988; Hullar and Minor, 1999), including high levels of spontaneous discharge around which modulation was induced by turntable rotation. It is important to note that in our recording configuration the animals’ heads were held in a fixed position (15° nose down, 10° recorded-ear down) so that turntable rotation evokes a clear modulation in afferents projecting from both horizontal and superior semicircular canal cristae. Even afferent discharge modulated by 1–2 spikes⋅s^-1^ are unambiguously identified and characterized with these methods and subsequent analyses. In untreated specimens, a relatively small fraction of afferents was encountered in each preparation whose discharge did not modulate with turntable rotation, which was consistent with afferents projecting from the utricle. These afferents were not further explored as our experimental configuration precluded the application of linear acceleration stimuli, even in a paradigm of eccentric rotation. Furthermore, since the head was held in a static tilt, resultant discharge from most utricular afferents could not be considered “spontaneous,” their discharge reflecting head position in a static tilt. These data indicated that when recording from the site described above in section “Materials and Methods” the majority of afferents encountered projected from the cristae, while only approximately 20% projected from the utricle. This observation is important in the context of evaluating afferents in gentamicin-treated specimens.

The balance of afferents probed at the recording site exhibited discharge that was clearly modulated during turntable rotations, as exemplified in **Figure 2B**. The afferents from *Untreated* preparations were selected to illustrate the high fidelity of responses to sinusoidal stimuli despite the low sensitivity measures (e.g., ±5 spikes⋅s^-1^ during sinusoidal stimuli of ±30°⋅s^-1^ peak velocity). In addition, robust stimulus-evoked responses were also recorded from superior and horizontal semicircular canal afferents in *Vehicle Control* specimens (right). Like the *Untreated* specimens, semicircular canal afferents in these controls were clearly modulated in response to sinusoidal turntable rotation, indicating that perilymphatic port implantation and infusion of HBSS did not lead to deleterious outcomes with respect to afferent electrophysiologic measures. High measures of stimulus–response coherence (>0.85) were observed in these two afferent groups, even under conditions of modest discharge modulation (±5 spikes⋅s^-1^). Measures of stimulus–response coherence remained high in these controls.

### Early Periods Following Gentamicin Administration Reveal Independence of Hair Cell and Afferent Neuron Pathotypes

Lesions to the peripheral vestibular receptors may involve hair cells, support cells, and afferent dendrites, inducing alterations in cellular function that may eventually lead to pathophysiologic changes in afferent discharge transmitted to the central nervous system. We refer to each general morphologic and physiologic component of the lesion as a *pathotype*, representing the different forms or “types” of pathology to specific components within the sensory epithelia. The goal of the present investigation was to refine production of the lesions so that unique pathotypes could be distinguished. Rather than strictly examine the lesions at the final mature stage, we envisioned that hair cell and afferent pathotypes might be distinguished if they exhibited differential temporal sensitivities to low-dose gentamicin. Therefore, we examined specimens at 0.5 and 1 months following gentamicin administration.

**FIGURE 3:**
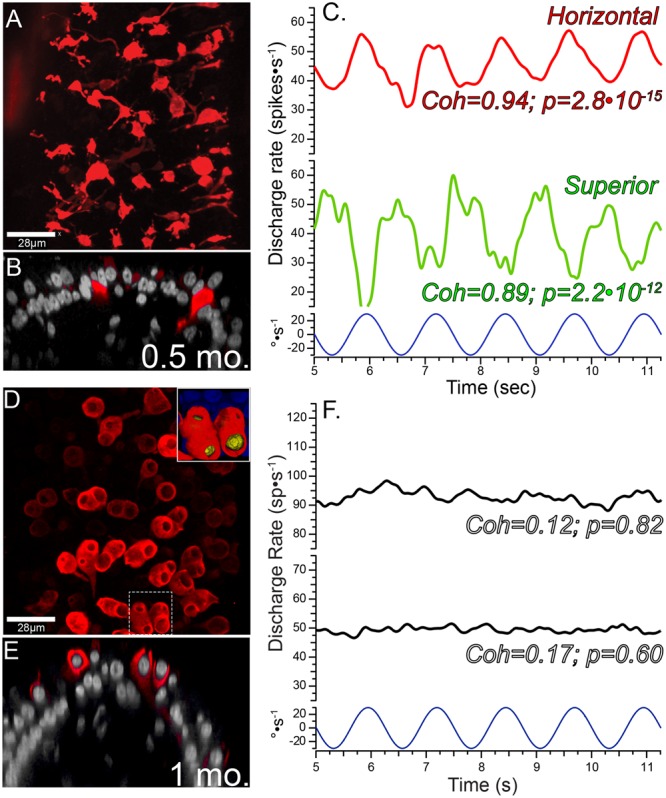
Pathophysiologic correlates in the 1st month following intraperilymphatic gentamicin administration. Micrographs of horizontal crista epithelia at 0.5 **(A,B)** and 1 **(D,E)** month following gentamicin administration illustrate the variability in the histopathology at these early post-administration periods. The discharge of two afferents from each preparation are shown to demonstrate the physiologic status of each (i.e., **C** corresponds to the micrographs in **A** and **B**; **F** corresponds to **D** and **E**). **(A,B)** Maximum intensity projection **(A)** and orthogonal optical section **(B)** views of CALB2+ afferents in the horizontal crista central zone 0.5 months after 1 μg gentamicin administration. All calyces retracted in this specimen, while remnant CALB2+ endings of parent axons and fine branches are observed. **(C)** In this preparation both horizontal and superior afferents exhibited robust responses to rotational stimuli despite the widespread central zone calyx retraction and very modest hair cell loss in this specimen (not shown). The 0.8 Hz stimulus trajectory (±30°⋅s^-1^) is represented by the bottom trace (blue). The coherence measures (*Coh*) demonstrated the high fidelity between stimulus and response that was retained at this early post-administration time. **(D,E)** Maximum intensity projection **(D)** and orthogonal optical section **(E)** views illustrating CALB2+ calyces in a horizontal crista central zone from a specimen harvested 1 month following 1 μg intraperilymphatic gentamicin. The fluted and narrow apical extensions seen in untreated calyces (see **Figure 1**) were absent in the majority of these calyces and appear to have retracted to exhibit wide openings in this treated specimen. This is illustrated in the orthogonal view **(E)** illustrating a calyx whose apical portion – just higher than the hair cell nucleus – is not observed. A 3D volume reconstruction of the boxed region (**D**, bottom) is shown at higher magnification in the inset (top right in **D**), for which the hair cell nuclei within the CALB2+ calyces were clearly visible through the widened apical openings of the calyces (recolored yellow). **(F)** Representative instantaneous discharge traces for two afferents recorded from the preparation represented in **(D,E)**. These afferents were unresponsive to the sinusoidal rotation (0.8 Hz, 30°⋅s^-1^, bottom), confirmed by the low coherence values (*Coh*) shown for each perstimulus discharge record.

The early post-administration periods provided insight into the progression of the lesions to the stable state observed at later post-administration times. The morphologic and physiologic pathotypes of specimens analyzed at 0.5 and 1 months are represented in **Figure 3**, and illustrate the heterogeneity in calyx morphology and associated physiology observed at these times. The morphologic and physiologic data shown in these figures were selected to be representative of the specimens, and direct associations between the afferent discharge records and the crista region highlighted in the micrographs cannot be made (i.e., without intracellular labeling and tract tracing). **Figures 3A,B** show maximum intensity projection and orthogonal views (respectively) of the horizontal crista from a 0.5-month specimen exhibiting severe pathology to CALB2+ calyces. All CALB2+ calyces in this specimen were retracted, characterized by CALB2+ parent axons without calyces or drastically malformed partial calyces (**Figure 3B**). The central zones of the three cristae from this specimen exhibited a mean decrease in hair cell density of approximately 24%. In the chinchilla crista central zone 60% of hair cells are type I (Desai et al., 2005a), indicating that even if the hair cell loss was restricted to type I in this specimen a large fraction of the phenotype remained. Therefore, while the lesion involved both hair cells and afferent calyces, the pathology to central zone CALB2+ calyces was more severe than central zone hair cell loss.

The discharge characteristics of afferents projecting to cristae of the preparation represented in **Figures 3A,B** are illustrated in **Figure 3C**, where the instantaneous discharge of afferents projecting to the horizontal and superior cristae during sinusoidal turntable velocity (**Figure 3C**, bottom) is shown. These records demonstrated that despite the histopathology exhibited by the crista epithelia (i.e., complete central zone calyx retraction and decreased hair cell density) afferent discharge was clearly modulated in response to sinusoidal stimuli. Additionally, stimulus-evoked discharge modulation (approximately ±7–10 spikes⋅s^-1^) is superimposed upon spontaneous discharge (40–45 spikes⋅s^-1^), indicating that spontaneous discharge is preserved in these specimens that exhibited significant pathology. Coherence measures were comparable to that observed in untreated and vehicle control specimens (**Figure 2B**).

The histologic and physiologic results from a preparation examined at 1 month post-administration is shown in **Figures 3D–F**, illustrating a unique histopathologic variant while the physiology of vestibular afferents demonstrated more severe functional compromise than shown in **Figure 3C**. Two of the three 1-month preparations exhibited complete calyx retraction, similar to that depicted by the micrographs in **Figures 3A,B**. The third preparation is shown in **Figures 3D,E**, illustrating CALB2+ calyces within the horizontal crista central zone that have lost the narrow fluted necks characteristic of untreated central zone calyces (**Figure 1**). The apical openings of most calyces in this specimen were widened. The inset micrograph, showing a volume reconstruction of the area enclosed by the dashed box (**Figure 3D**, bottom), illustrates that the calyx opening was sufficiently large to enable visualization of the enclosed hair cell nuclei (yellow). Because these calyces extended above the hair cell nucleus, they were counted as intact calyces (i.e., conforming to the specified criteria) despite the obvious morphologic anomaly.

The consistent electrophysiologic feature among all afferents recorded from 1-month post-administration preparations was the absence of responses to sinusoidal rotation. These are represented by the instantaneous discharge records shown in **Figure 3F**, which were recorded from the same preparation that yielded **Figures 3D,E**. These traces are shown to be representative of the unresponsive afferents recorded in this preparation, and they are not meant to be directly associated with the horizontal crista represented in the micrograph. The absence of stimulus-evoked discharge modulation precludes us from drawing the direct correlate. The stimulus–response coherence measures are shown for each trace, indicating the absence of discharge modulation to the stimulus (**Figure 3F**, bottom). However, these afferents exhibited robust spontaneous discharge. This preparation is remarkable in that full calyx retraction had yet to dominate the crista landscape, yet afferent discharge reflected the severe response attenuation characteristic of other specimens evaluated at 1 month post-administration (e.g., **Figure 7F**). This contrasts the clear stimulus-evoked discharge modulation in the 0.5 month specimen recorded in afferents projecting to cristae where central zone calyces have fully retracted (**Figures 3A–C**). These observations supporting the conclusions that severe attenuation in stimulus-evoked afferent discharge modulation did not depend upon full calyx retraction, and that these two pathotypes are associated with independent mechanisms.

### Sensory Dysfunction Appears Permanent

**FIGURE 4:**
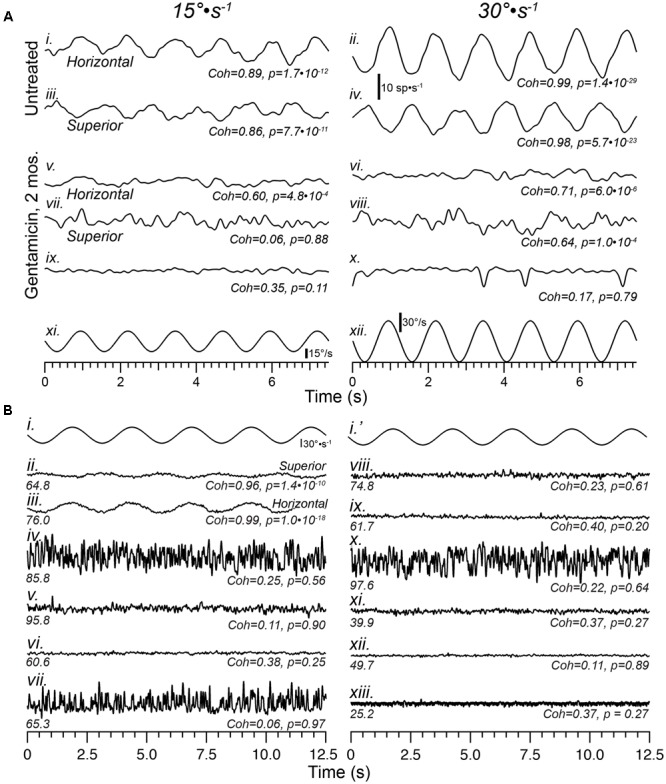
Sensory function is severely compromised by 2 months, and continues through 6 months, following intraperilymphatic gentamicin. **(A)** Representative responses of horizontal and superior semicircular canal afferents recorded from Untreated **(i–iv)** and gentamicin-treated **(v–x)** specimens in response to 0.8 Hz sinusoidal rotations of 15 (left) and 30°⋅s^-1^ (right) peak velocity (bottom traces in **A**). Among afferents recorded in three preparations at 2 months post-administration, most exhibited no response to rotation, similar to the traces in **(ix,x)** (verified by the low coherence values at each stimulus magnitude). However, in the gentamicin-treated preparation represented in this figure meager response were recorded in a few horizontal and superior afferents, shown in traces labeled **(v–viii)**. Though the coherence values were lower than those typically measured in untreated preparations, the responses were verified for the horizontal afferent at both stimulus magnitudes, and the superior afferent only at 30°⋅s^-1^ magnitude. The third afferent from this preparation was unresponsive **(ix,x)**. The discharge rate scale shown beneath the untreated horizontal afferent (30°⋅s^-1^) applies to all traces in **(A)**. **(B)** Instantaneous discharge records of afferents during sinusoidal rotation (0.4 Hz, 30°⋅s^-1^; **i,i′**) obtained 6 months following gentamicin administration are shown. For comparison, representative perstimulus discharge records of afferents projecting from the superior **(ii)** and horizontal **(iii)** cristae in an untreated specimen are shown. The mean spontaneous discharge rates are provided at the left underneath each record, while stimulus–response coherence measures (*Coh*) and corresponding *p*-values (*p*) are shown at right. Note the high coherence values for afferents from the untreated specimen, even the superior afferent in which the stimulus-evoked modulation is very modest. The 10 afferents from the treated specimen **(iv–xiii)** were recorded in succession during the experimental session, and all exhibited low coherence measures that corresponded with probabilities greater than 0.2 that this measure could have arisen randomly. The low coherence measures confirmed that these afferents were unresponsive to the sinusoidal rotational stimulus. See text for more detailed interpretation.

As illustrated in **Figure 3**, stimulus-evoked modulation was severely attenuated in all afferents recorded 1-month following gentamicin administration. **Figure 4** demonstrates that this sensory dysfunction persists into post-administration times of 2 and 6 months. Detailed responses of afferents recorded from a 2-month preparation are shown in **Figure 4A**, illustrating paired perstimulus discharge from the same afferents during presentation of 0.8 Hz rotational sinusoids at 15 (left: **Figures 4A-i,iii,v,vii,ix**) and 30°⋅s^-1^ (right; **Figures 4A-ii,iv,vi,viii,x**) peak stimulus velocities. The trajectories of these stimuli are illustrated by the bottom waveforms (**Figures 4A-xi,xii**), and representative responses of horizontal (**Figures 4A-i,ii**) and superior (**Figures 4A-ii,iv**) semicircular canal afferents from untreated specimens are shown at the top. The responses of three afferents from the same preparation are shown in **Figures 4A-v–A-x**, and illustrate the heterogeneity in the severe compromise found in this preparation. The histopathology of this horizontal crista is illustrated in **Figure 7G**, where complete loss of CALB2+ calyces was observed. **Figures 4A-v,vi** represent the discharge of a horizontal canal afferent exhibiting severely compromised responses to both 15 and 30°⋅s^-1^ stimulus intensities, but coherence analyses confirmed statistically verifiable responses (*p* = 4.8⋅10^-4^ and 6.0⋅10^-6^, respectively). The perstimulus discharge records of the superior canal afferent (**Figures 4A-iii,vii**) illustrated the case where coherence analysis indicated the absence of a response in the discharge at 15°⋅s^-1^, but the discharge during the 30°⋅s^-1^ peak velocity stimulus did evoke a verifiable response (*p* = 1.0⋅10^-4^). The perstimulus discharge of the third afferent (**Figures 4A-ix,x**), however, did not exhibit modulation by either stimulus magnitude (*p* = 0.11). These data demonstrate that severe response attenuation, and not solely response elimination, was a potential outcome of gentamicin administration at this post-administration time. In addition, these data illustrate the value in computing stimulus–response coherence as a firm metric to analyze these severely attenuated responses.

The physiologic ramifications of intraperilymphatic administration of 1 μg gentamicin at 6 months post-administration are shown in **Figure 4B**. These data depict 12.5 s epochs of perstimulus discharge (in spikes⋅s^-1^) from 10 afferents recorded consecutively in a single preparation during presentation of 0.4 Hz sinusoidal rotations (30°⋅s^-1^ peak velocity; **Figures 4B-i,i**′). For reference, representative response discharge recordings from superior and horizontal semicircular canal afferents in an untreated preparation at this stimulus intensity are shown in **Figures 4B-ii,iii**, respectively. The coherence values associated with these two responses were 0.96 and 0.99, with associated *p*-values of 1.4⋅10^-10^.and 1.0⋅10^-18^, respectively. Particularly notable is the high coherence of the superior canal afferent (**Figures 4B-ii**) despite the very low sensitivity and small modulation depth, indicating the coherence measures remain very high even in afferents for which the discharge modulation is extremely low.

The discharge characteristics representative of afferents recorded 6 months after receiving 1 μg intraperilymphatic gentamicin are represented in **Figures 4B-iv–B-xiii**, illustrating the perstimulus discharge during 0.4 Hz rotation (30°⋅s^-1^ peak velocity, **Figures 4B-i,i**′; the same stimulus is shown in the interest of providing temporal correlation with the discharge records beneath). These data demonstrate three fundamental features of the electrophysiologic pathotype. First, coherence analyses verify the absence of rotational responses in all 10 perstimulus discharge records (Coh < 0.38; *p* > 0.2). While some of these afferents may have projected to the utricle (which, under normal conditions, would be unresponsive to rotation), the probability that all ten afferents would project to the utricle is extremely low. Therefore, the absence of a response in all 10 afferents further verifies the severe response attenuation among afferents from treated labyrinths. Second, the compromise of response capabilities among semicircular canal afferents, first observed at 1 month post-administration, persisted through 6 months post-administration, which provided evidence indicating the vestibular sensory epithelia do not exhibit capabilities for spontaneous recovery by 6 months. Third, despite the severe response attenuation spontaneous discharge remained robust at this post-administration time, with some afferents exhibiting rates exceeding 80 spikes⋅s^-1^ (e.g., **Figures 4B-iv,v,x**). This demonstrated an absence of any progressive compromise in spontaneous discharge through the 6 month post-administration interval.

### Alterations in Spontaneous Discharge Associated with Intraperilymphatic Gentamicin

The principal finding from electrophysiologic recordings of vestibular afferents following intraperilymphatic administration of 1 μg gentamicin was the persistence of spontaneous discharge while modulation in response to head movement rotations was severely attenuated or lost. We tested the null hypothesis that spontaneous discharge characteristics of afferents recorded from gentamicin-treated preparations were similar to those of our laboratory’s database of semicircular canal afferents recorded from untreated preparations. We also conducted parallel analyses of previously published spontaneous discharge data recorded from normal (untreated) semicircular canal and utricular afferents (Baird et al., 1988; Goldberg et al., 1990). These latter analyses provided an independent context for comparing distributions of spontaneous discharge characteristics derived from rotationally sensitive (semicircular canal) and rotationally insensitive (utricular) afferents, similar to the classifications of untreated semicircular canal (rotationally sensitive) and gentamicin-treated (rotationally insensitive) afferents.

**FIGURE 5:**
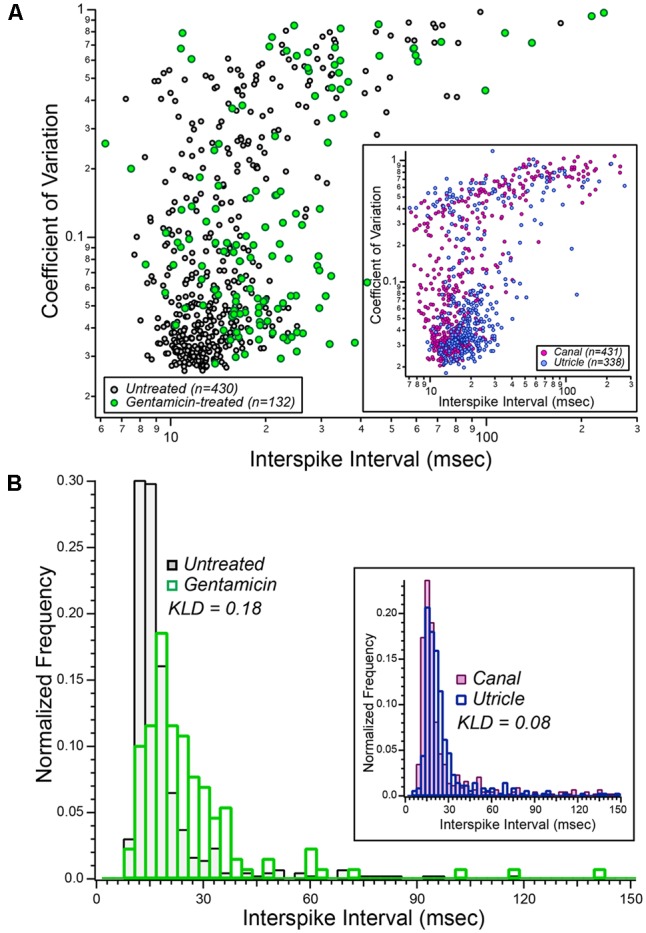
Aggregate distributions of spontaneous discharge characteristics in vestibular afferents recorded after low-dose gentamicin administration. **(A)** Scatterplot illustrating the comparison of spontaneous discharge characteristics from gentamicin-treated specimens (green symbols) and the laboratory’s database of 430 semicircular canal afferents from normal (untreated) specimens (black/gray symbols). These data illustrated that the mean spontaneous ISIs among afferents from gentamicin-treated preparations appeared to be greater (right-shifted in this scatter plot) than afferents recorded from untreated specimens. These data are interpreted in the context of published data from semicircular canal (Baird et al., 1988) and utricular (Goldberg et al., 1990) afferents, shown in the inset, representing a comparison of spontaneous discharge characteristics from canal and utricle afferents recorded from the same laboratory, and therefore under similar conditions. **(B)** Normalized histograms of mean ISIs from gentamicin-treated (green bars, *n* = 132) and untreated (black/gray bars, *n* = 430) preparations (i.e., mean ISI data from **A**). The Kullback–Leibler divergence between these two distributions was 0.18, and resampling analyses indicated the probability that this KLD could have been derived from random sampling of a single combined distribution was less than 10^-6^ (i.e., *p* < 10^-6^). The inset histograms were derived from the published mean interspike interval (ISI) data of semicircular canal and utricular afferents (i.e., from inset in **A**). The KLD for these distributions was 0.08, reflecting the expected difference in mean intervals between canal and utricular afferents from the same preparations. See text for details.

The spontaneous discharge characteristics of afferents from gentamicin-treated preparations (green symbols) are shown in **Figure 5A**, against a backdrop of the characteristics obtained from our database of untreated semicircular canal afferents (black/gray symbols). The inset plot illustrates previously published spontaneous discharge data of semicircular canal and utricular afferents (Baird et al., 1988; Goldberg et al., 1990), reconstructed as described in section “*Materials and Methods*.” The distribution of gentamicin-treated afferents appears to be right-shifted in the main plot, suggesting that the mean ISIs from this afferent group are, collectively, longer than untreated semicircular canal afferents. A similar impression is also given by the inset scatterplot, in which the cloud of utricular afferents appears to represent slightly greater mean ISIs. To compare the distributions of mean ISIs among untreated and gentamicin-treated populations (and between previously published semicircular canal and utricular afferents), histograms were prepared and are shown in **Figure 5B** (inset), in which the longer mean ISIs of gentamicin-treated afferents are visualized. The two distributions were compared by computing the KLD, after which a resampling strategy was implemented to test the hypothesis that the mean ISI distributions were derived from random sampling from a single underlying distribution. This probability was less than 1.0⋅10^-6^, supporting rejection of the null hypothesis. Furthermore, this result indicated that the distribution of mean ISIs from our sample of gentamicin-treated preparations corresponded to longer mean intervals compared to those of untreated semicircular canal afferents.

The histograms within the inset of **Figure 5B** represent the distributions of ISIs for semicircular canal and utricular afferents previously reported (Baird et al., 1988; Goldberg et al., 1990). These data make an important contribution to the interpretation of the data reported herein in that they represent the expected differences in spontaneous discharge characteristics between semicircular canal and utricular afferents. The computed KLD between these distributions was 0.081, and resampling analyses supported the conclusion that the mean intervals for utricular afferents were longer than that of semicircular canal afferents (*p* = 2⋅10^-6^). The difference in mean interval distributions between semicircular canal and gentamicin-treated afferents (KLD = 0.18) was more than twice that expected between semicircular canal and utricular afferents from the same preparations. This finding supports the conclusion that the difference in mean ISI distributions of untreated and gentamicin-treated preparations is much greater than that expected from canal-utricle differences.

Spontaneous discharge coefficient of variation (CV) was also compared between untreated and gentamicin-treated distributions (KLD = 0.080), and between distributions of previously published data from semicircular canal and utricular afferents (KLD = 0.040). Through resampling analyses, we concluded that the CVs of gentamicin-treated afferents were greater than that of untreated afferents (*p* = 2.6⋅10^-4^). However, similar resampling analyses showed that the CVs of previously published data from semicircular canal and utricular afferents were similar (*p* = 0.34). This latter analysis further supports the conclusion that the difference in CV distributions between gentamicin-treated and untreated afferents cannot be explained by a mis-categorization of rotationally insensitive utricular afferents as a characteristic of a gentamicin-treated pathotype.

### Morphologic Lesions Associated with Severe Functional Deficit

**FIGURE 6:**
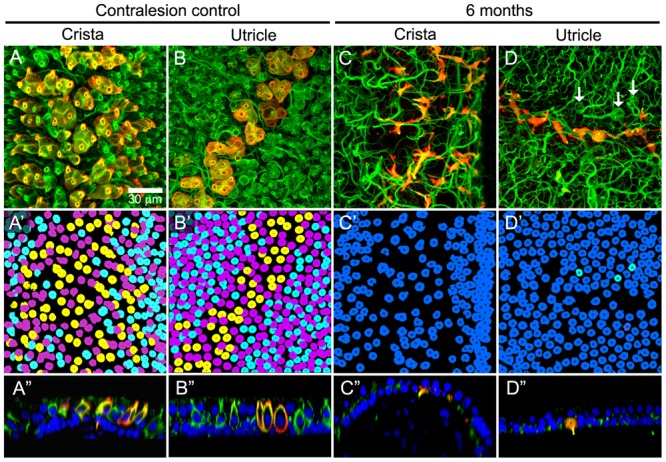
Extensive calyx loss is seen in vestibular sensory epithelia exposed to 1 μg gentamicin. Horizontal semicircular canal cristae and utricles were immunolabeled with anti-CALB2 (red) and anti-TUBB3 (green) antibodies, and nuclei were labeled with a Nissl stain. **(A–D)** Confocal projections illustrating afferent calyces projecting to the cristae central-intermediate zones and utricle peristriola regions in contralesion control **(A,B)** and gentamicin-treated **(C,D)** specimens 6 months post-administration. Only three TUBB3+ calyces (arrows) can be seen in the micrograph of the treated utricle **(D)**. **(A′–D′)** Projections of hair cell nuclei of the neuroepithelial areas shown in **(A–D)**. Nuclei are colored in **(A′,B′)** to indicate type I_c_ (yellow), type I_d_ (cyan), and type II (magenta) hair cells. In the treated epithelia **(C′–D′)** most hair cell types were indistinguishable because of extensive calyx loss, and nuclei are colored to indicate uncharacterized (blue) and type I_d_ (cyan) hair cells. **(A″–D″)** Orthogonal optical sections illustrate diminished laminar organization of sensory and support cell nuclei (Nissl stain; blue) in gentamicin-treated specimens. Scale shown in **A** applies to all panels.

Intraperilymphatic administration of 1 μg gentamicin resulted in lesions of the vestibular epithelia characterized by afferent calyx retraction and modest hair cell loss. These pathotypes are illustrated in the maximum intensity projection micrographs of horizontal canal cristae (central zone) and utricles (peristriola) from contralesion control (**Figures 6A,B**) and gentamicin-treated (**Figures 6C,D**) specimens immunolabeled with anti-CALB2 (red) and anti-TUBB3 (green). In the contralesion control specimens (**Figures 6A,B,A″,B″**), anti-TUBB3 immunolabeling (TUBB3+) extends into the calyx neck as was found for the anti-CALB2 labeling (see also **Figure 1**). The CALB2+ calyx defined type I_c_ hair cells [i.e., those that received a CALB2+ calyx and associated with calyx-only afferents, (Li et al., 2008)], while the balance of type I hair cells associated with TUBB3+ calyces that were CALB2 negative represent type I_d_ [i.e., associated with dimorphic afferents (Li et al., 2008)]. This provided positive criteria to identify Nissl-stained hair cell nuclei belonging to types I_c_, I_d_, or II (i.e., hair cell nuclei not associated with a calyx) hair cells. Hair cell nuclei within the contralesion control confocal stacks that were identified in this way are shown in **Figures 6A′,B′** in yellow (type I_c_), cyan (type I_d_), or magenta (type II), providing a graphic illustration of hair cell density in the crista central zone and utricular peristriola.

The gentamicin-treated specimens exhibited sharp contrast to the contralesion control specimens as illustrated by the micrographs of horizontal crista and utricle specimens harvested 6 months after gentamicin administration (**Figures 6C,C′,C″,D,D′,D″**). Virtually all calyces (i.e., both CALB2+ and TUBB3+) are lost in the gentamicin-treated specimens, though CALB2+ and TUBB3+ parent axons remain within the neuroepithelia (**Figures 6C,D,C″,D″**). The absence of calyces and preservation of parent axons indicates a pathology that predominantly involved the calyx, which we refer to as *calyx retraction*. The extensive retraction was apparent for both CALB2+ and TUBB3+ calyces, indicating that CALB2+ calyx retraction is representative of all calyces at this post-administration time. **Figures 6C′,D′** illustrate the spatial density of hair cell nuclei, which was lower (i.e., more empty space) than that found in the contralesion control specimens (**Figures 6A′,B′**) and indicated partial hair cell loss. Because of extensive calyx loss and thinning of the epithelia, it was impossible to determine hair cell phenotype based on calyx innervation pattern or laminar organization of nuclei, except in the rare occurrence when partial calyces persisted (**Figure 6D′**, cyan nuclei). Therefore, the micrographs in **Figures 6C′,D′** illustrate all unsegregated hair cells.

**FIGURE 7:**
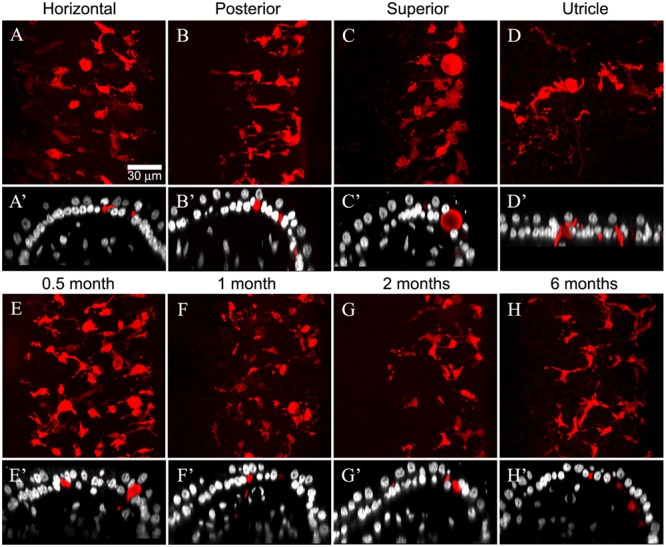
Gentamicin induced damage is similar across vestibular endorgans and at all post-administration time periods. Damage as illustrated by CALB2 immunoreactivity (red) and Nissl stain (grayscale) for endorgans of a single animal 2 months after administration **(A–D)** and in horizontal cristae ampullaris 0.5, 1, 2, and 6 months after exposure **(E–H)** is shown. Maximum intensity projections of confocal image stacks taken from the central-intermediate zone of the cristae and the peristriola region of the utricle demonstrate extensive CALB2+ calyx loss in all endorgans **(A–D)** and at all time periods **(E–H)**. Orthogonal optical sections illustrate similar morphological characteristics in all endorgans **(A′–D′)** and at all time periods **(E**′**–H**′**)**. Scale shown in **A** applies to all micrographs.

The cristae from gentamicin-treated specimens were subject to histologic examination at 0.5, 1, 2, or 6 months post-administration times. At each interval, we found the effects of 1 μg gentamicin to be comparable across individual epithelia within a given labyrinth. This is illustrated in **Figures 7A–D** for one preparation analyzed 2 months following gentamicin treatment. These micrographs, representing maximum intensity projections of anti-CALB2 immunoreacted specimens, illustrate the retraction of all CALB2+ calyces in the cristae and utricle from a single labyrinth. Representative orthogonal sections are shown in **Figures 7A**′–**D**′. **Figures 7E–H** illustrate the time course of calyx retraction in horizontal cristae at the indicated post-administration times. Complete calyx retraction (CALB2+ and TUBB3+) was seen in 2 of 3 specimens examined at 0.5 and 1 month post-administration. Intact calyces were extremely rare at 2 and 6 months post-administration (shown for CALB2-immunoreacted specimens in **Figures 7G–H**). The morphologies of 0.5- and 1-month specimens were more variable; and while evidence of the lesion was observed in all specimens, some exhibited partially retracted calyces rather than complete retraction (see **Figure 3D**). At post-administration durations of 2–6 months, the majority of CALB2+ parent axons terminated as blunt endings within the sensory epithelia.

### The Majority of Calyces Retracted While Most Hair Cells Survived Exposure to 1 μg Gentamicin

#### Calyces and Parent Axons

**FIGURE 8:**
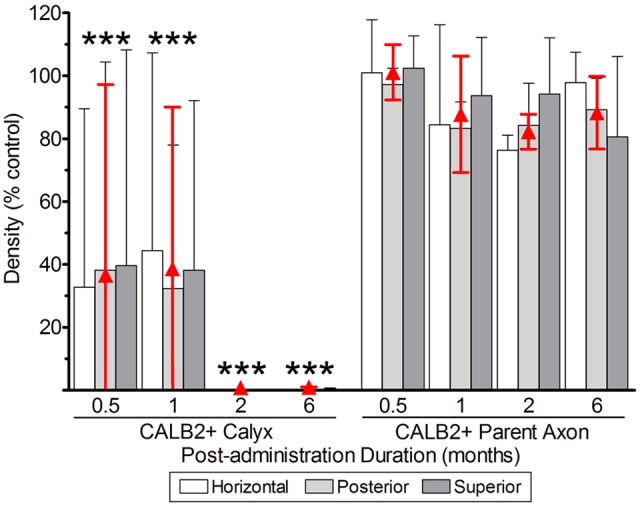
CALB2+ afferent damage after 1 μg gentamicin administration. Normalized CALB2+ calyx and parent axon densities, expressed as percentages of contralesion controls, are shown for horizontal, posterior and superior cristae at the specified post-administration times (i.e., 0.5, 1, 2, and 6 months). ANOVAs indicated the absence of a main effect for crista type, and therefore the mean densities across all cristae are represented by the red triangles. All data are shown as mean ± SD. Triple asterisks (^∗∗∗^) correspond to *p* < 0.001.

**FIGURE 9:**
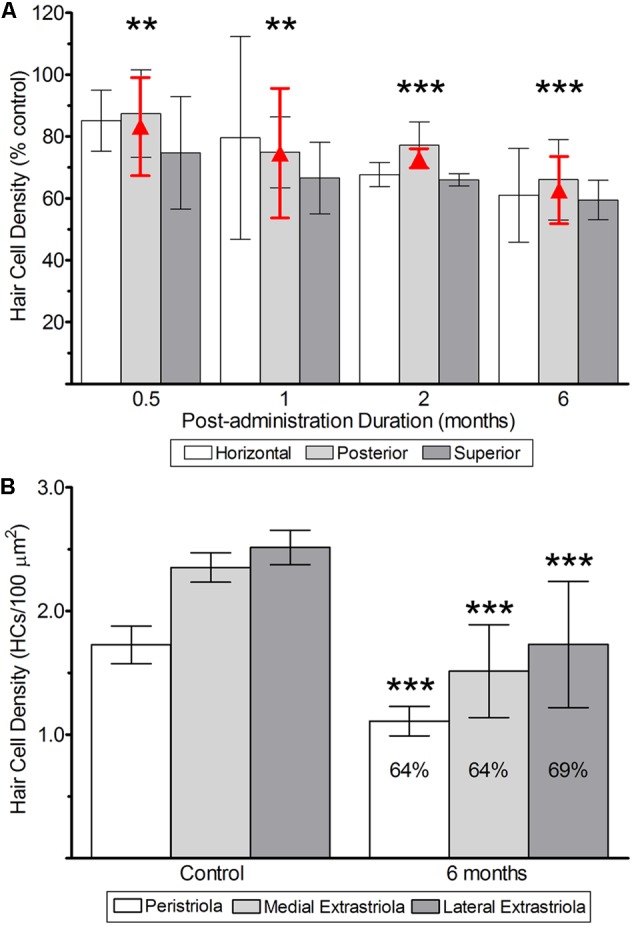
Hair cell survival after 1 μg gentamicin administration. **(A)** Hair cell survival in cristae from labyrinths treated with 1 μg gentamicin. As noted above, two-factor ANOVA revealed the absence of a main effect of crista type, and therefore the mean densities (±*SD*) are represented by the red triangles (and error bars). **(B)** Hair cell survival in utricles determined 6 months after gentamicin administration. The mean hair cell survival at this post-administration time in the peristriola (64%), medial extrastriola (64%), and lateral extrastriola (69%) were similar. All data are shown as means (±*SD*). Double asterisks (^∗∗^) correspond to *p* < 0.01; triple asterisks (^∗∗∗^) correspond to *p* < 0.001.

The quantifications of CALB2+ calyces, parent axons, and hair cells reflecting lesions to vestibular epithelia (**Figures 6, 7**) are illustrated in **Figures 8** and **9**, and summarized in **Tables 1** and **2**. The effects on CALB2+ calyces and parent axons are shown in **Figure 8**, depicting their densities at all post-administration times. We found only 36.9 ± 63.87% (*n* = 3; *p* < 0.001) and 38.80 ± 63.87% (*n* = 3; *p* < 0.001) of CALB2+ calyces remained 0.5 and 1 month after gentamicin exposure, respectively; however, nearly all CALB2+ calyces had retracted by 2 and 6 months post-administration (*p* < 0.001; **Figure 8**). No differences between cristae (i.e., horizontal, superior, or posterior) harvested at the same post-administration duration were found (*p* > 0.05). Although calyces were found in some samples, no complex calyces were observed in damaged specimens with the exception of two; a 0.5-month specimen that showed no signs of morphological damage or hair cell loss, and a 1 month specimen that exhibited a more modest lesion than other comparably treated specimens with reduced hair cell density and malformations in calyx structure (i.e., calyces without necks and retracted calyces; **Figure 3D**). These specimens came from preparations made very early in our experience, and it is possible that they reflect incidences of incomplete gentamicin infusion.

Although most calyces within gentamicin-treated labyrinths retracted, the majority of CALB2+ parent axons remained at the sensory epithelium at all post-administration periods, supported by the finding that parent axon densities were not different from contralesion controls (*p* > 0.05; **Figure 8**). Damaged afferents (including CALB2+ dendrites and intermediate diameter TUBB3+ fibers without calyceal endings) remained at the sensory epithelia at least 6 months after gentamicin exposure (also represented in **Figure 6**). As illustrated in **Figure 8**, it was estimated that the fractions of CALB2+ parent axons (expressed as percent of contralesion control) present at the sensory epithelia were 100.0 ± 9.3 (0.5 months; *N* = 3), 87.2 ± 19.56 (1 month; *N* = 3), 83.0 ± 5.87 (2 months; *N* = 3) and 87.0 ± 12.19 (6 months; *N* = 4).

#### Hair Cells

The quantification of hair cell densities required a critical evaluation of lesioned epithelia to distinguish hair cell and support cell nuclei. Hair cell nuclei were identified predominantly by morphology and chromatin condensation as previously described. In general hair cell nuclei remained located more apical-ward in gentamicin-treated epithelia as found in untreated specimens, though this distinction became less clear in damaged specimens. In addition, hair cell nuclei often exhibited asymmetrical morphologies after gentamicin exposure. These variants were most pronounced in specimens with the heaviest damage as characterized by gross morphological changes and hair cell loss. For these specimens, a qualitative analysis of chromatin density became an important identifying criterion for hair cell nuclei.

The measures of hair cell densities following low-dose gentamicin administration portray a different picture than that of the afferents. This is illustrated in **Figure 9A**, showing densities of hair cell nuclei in the central zone of vertical and horizontal cristae at all post-administration durations, and in **Figure 9B** for the utricular striola at 6 months post-administration. The low-dose gentamicin exposure induced hair cell loss in vestibular epithelia at all post-administration periods. As shown for the calyx and parent axon counts, hair cell densities are expressed as percentages of contralesion controls (**Figure 9**) and counts per 100 μm^2^ (**Table 2**). The central zones of horizontal, superior, and posterior cristae from treated specimens exhibited similar hair cell densities at all post-administration durations (**Table 2**), indicating that they exhibited comparable hair cell loss. We determined that over 60–80% of central zone hair cells survived following 1 μg gentamicin exposure (**Figure 9A**), where hair cell survival in pooled cristae (mean % of contralesion control, shown as red symbols, ±standard deviation) was 82.5 ± 14.01 at 0.5 months (*p* < 0.01), 73.7 ± 18.62 at 1 month (*p* < 0.01), 71.1 ± 2.68 at 2 months (*p* < 0.001) and 61.6 ± 9.55 at 6 months (*p* < 0.001). In addition, hair cell densities were measured in the utricular peristriola, as well as from lateral and medial extrastriola regions for 6 month specimens [the post-administration duration that exhibited the most damage (**Figure 9B**)]. Control specimen hair cell density (hair cell nuclei per 100 μm^2^) of the peristriola region was estimated at 1.7 ± 0.15, and 2.5 ± 0.14 and 2.4 ± 0.12 for the lateral and medial extrastriola regions, respectively (*N* = 4). In comparison, hair cell densities of the gentamicin treated specimens were 1.1 ± 0.12 for the peristriola region, and 1.7 ± 0.51 and 1.5 ± 0.38 for the lateral and medial extrastriola regions (*N* = 4; *p* < 0.001). The relative densities computed from these absolute densities were similar to the central zone cristae at 6 months post-administration, which amounted to 64, 64, and 69% for the peristriola, medial extrastriola and lateral extrastriola (respectively).

It has been previously reported that the thickness of the sensory epithelium (lumenal surface to the basement membrane) decreases as a consequence of the morphologic alterations induced by gentamicin (Forge et al., 1998). Therefore, we evaluated whether such volumetric modifications alone could account for the quantitative changes in hair cell, calyx, or axon densities. For example, if gentamicin-induced decreases in epithelial volume resulted in a concomitant decrease in epithelial area without loss of its constituents, the analytical tendency would be to increase the density of components within these volumes. We evaluated the effects of any putative volumetric changes caused by gentamicin exposure by estimating support cell densities in the horizontal semicircular canal cristae central zones for normal and treated specimens. We posited that support cells would be minimally affected by low-dose gentamicin exposure, and that if gentamicin-induced volume changes did result in decreased epithelial areas we would find that untreated normal specimens would exhibit lower support cell densities than the gentamicin-treated specimens. We found that support cell densities (support cells/100 μm^2^) were similar for the 0.5-month (2.1 ± 0.11; *N* = 3), 1-month (2.2 ± 0.01; *N* = 2), 2-month (2.1 ± 0.19; *N* = 3), 6-month (1.8 ± 0.18; *N* = 3), and normal (2.1 ± 0.19; *N* = 3) specimens. These data support the conclusion that the alterations in epithelia morphology concomitant with gentamicin treatment could not account for the quantitative changes in hair cell or CALB2+ afferent densities.

Previous assessments of the distribution of hair cell types (i.e., types I and II) indicated that type I hair cells represented approximately 60% of all hair cells within the central zones of chinchilla cristae (Desai et al., 2005a). The loss of all calyces and morphologic stereotypes precludes an evaluation of the remaining hair cell types in treated specimens. However, even if all lost hair cells were type I, the fact that the minimum mean fraction of surviving hair cells (i.e., at 6 months post-administration) was over 60% indicate that a notable number of type I hair cells survive the treatment that yields total loss of afferent calyces. Therefore, our hair cell density measures alone indicate that some type I hair cells remain viable – albeit functionally compromised – within the cristae central zones and utricular striolae in labyrinths exposed to 1 μg gentamicin, even when all calyces have retracted.

### Viable, Morphologically Altered Hair Cells at 6 Months Post-administration

**FIGURE 10:**
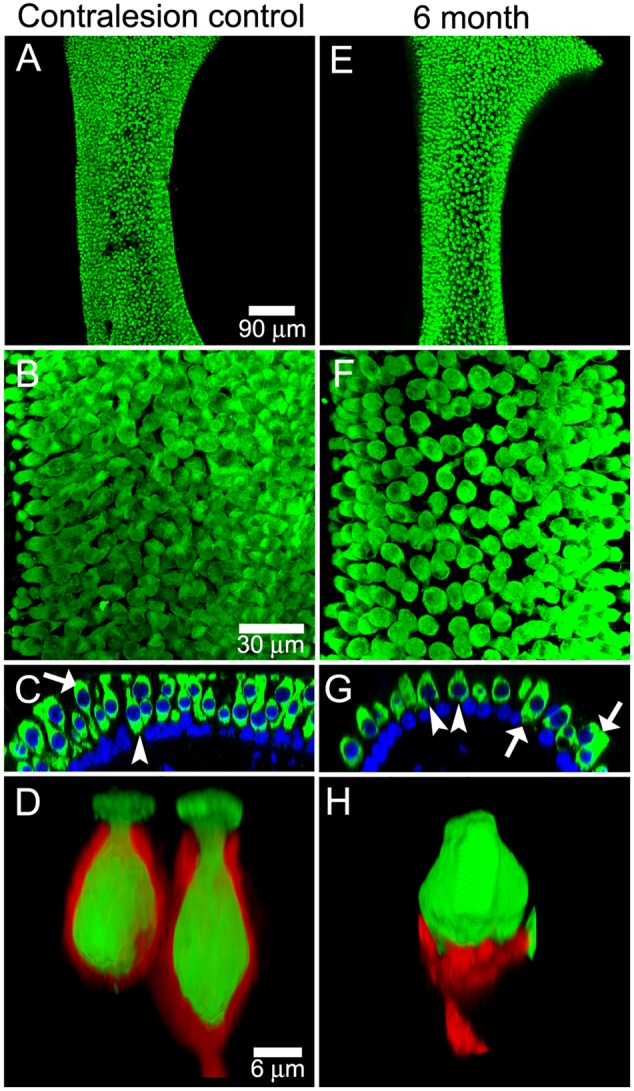
Surviving hair cells following gentamicin exposure exhibit aberrant morphology. Contralesion control **(A–D)** and gentamicin-treated **(E–H)** superior semicircular canal cristae were immunolabeled with anti-MYOVI (green) and anti-CALB2 (red) antibodies, and were Nissl-stained (blue) 6 months after gentamicin administration. **(A,E)** Low magnification confocal projections illustrating the distribution of MyoVI+ hair cells. **(B,F)** Higher magnification confocal projections images of the central-intermediate zones illustrate reduced MYOVI+ hair cell densities in the lesioned epithelia. **(C,G)** Orthogonal optical sections of the untreated and treated specimens. Cells with morphologies characteristic of type I (arrowhead) and type II (arrow) hair cells can be seen in an optical orthogonal section of the undamaged specimen **(C)**. However, hair cells with aberrant morphologies resembling “tear drops” (arrowheads) and “barrels” (arrows) are observed in treated specimens **(G)**. **(D,H)** 3D volume reconstructions show two MYOVI+ type I hair cells enveloped by a CALB2+ complex calyx in a contralesion control specimen **(D)**, and a hair cell exhibiting “tear-drop” morphology is shown closely-apposed to a CALB2+ dendrite in a gentamicin-treated specimen **(H)**. The CALB2 signal in **(D)** was made translucent to enable visualization of MYOVI signal. Scale bars are the same for **(A,E)** (90 μm), **(B,C,F,G)** (30 μm), and **(D,H)** (6 μm).

Anti-MYOVI and anti-CALB2 immunohistochemistry was used to visualize hair cell morphology and the association with intact and remnant CALB2+ calyces in contralesion-control and gentamicin-treated specimens. As shown for superior semicircular canal cristae in **Figure 10**, MYOVI immunoreactivity was robust in contralesion control hair cells that were tightly packed in the sensory epithelia (**Figures 10A,B**). Hair cells with type I (arrowhead; **Figure 10C**) and type II (arrow; **Figure 10C**) morphologies were easily recognizable in optical orthogonal sections of the untreated specimens. Most type II hair cells exhibited the stereotypical morphology being broad around the more apically located nuclei and narrow and cylindrical at their bases. Type I hair cells exhibited the prototypical “chalice” morphology with a thickened plate atop the thin apical neck. This is further illustrated in the reconstructions of two type I_c_ hair cells shown in **Figure 10D**, highlighted for comparison to hair cells from gentamicin-treated epithelia. In this reconstruction, the CALB2+ calyces (red) were made translucent for visualization of the encapsulated MYOVI+ hair cells.

The morphologic changes in hair cells resulting from low-dose gentamicin administration is shown in **Figures 10E–H**, representing the central zone of a superior crista 6 months post-administration. While plenty of viable hair cells are indicated by robust anti-MYOVI immunolabeling, they were less densely packed reflecting hair cell loss (**Figures 10E,F**). Hair cells exhibiting the stereotypical types I and II morphology were observed in few treated specimens (not shown); however, most hair cells lost distinguishing morphological characteristics as illustrated in the orthogonal optical section shown in **Figure 10G**. Some hair cells in damaged epithelia exhibited a “tear drop” morphology (arrowheads; **Figure 10G**) while others exhibited a “barrel” morphology (arrows; **Figure 10G**). The “tear drop” hair cells, although sharing similar morphological characteristics to type I hair cells, did not have the typical elongated neck of untreated type I hair cells. Some hair cells with ‘tear drop” morphologies were contacted by a retracted CALB2+ partial calyx as illustrated by the reconstruction in **Figure 10H**. In addition, partial calyces were observed to encapsulate only the bases of such “tear-drop” hair cells, inferring that hair cells with such morphologies are remnant type I_c_ hair cells.

### Fine Dendritic Projections Persist in Gentamicin-Treated Epithelia

**FIGURE 11:**
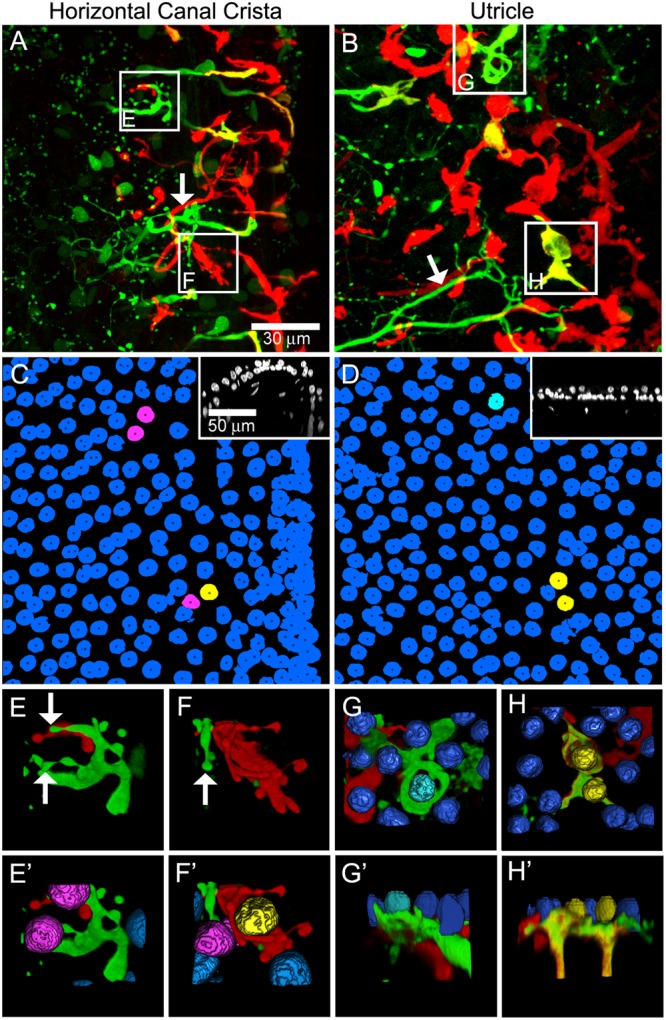
Biocytin labeled fibers illustrate patent small diameter dendritic branches and endings exhibiting bouton-like morphology in lesioned vestibular epithelia. Shown are micrographs of the horizontal semicircular canal crista and utricle from a specimen whose superior vestibular nerve was retrogradely labeled with TRITC-biocytin (green) 6 months post-administration. The specimens were also labeled with anti-CALB2 antibody (red) and Nissl stain (blue; nuclei in insets are grayscale). **(A,B)** Maximum intensity projections from the central and intermediate zones of the crista **(A)** and peristriola region of the utricle **(B)** illustrate the presence of biocytin-labeled fibers (arrows) projecting to the neuroepithelial regions harboring CALB2+ fibers. These fibers, which in untreated specimens would exhibit dimorphic dendritic morphologies (i.e., CALB2-negative fibers within these crista zones), were also without calyces as expected (see **Figure 6**). However, they do*(exhibit a number of putative boutons en passant, seen as varicosities along thin dendritic branches. **(C,D)** Maximum intensity projections of hair cell nuclei present in the confocal projections shown in **(A,B)**. Nuclei are colored to indicate representative hair cell nuclei closely apposed to a partially collapsed CALB2+ calyx (yellow in **C** and **D**), a partially collapsed biocytin-labeled calyx (cyan in **D**), and bouton-like endings (magenta in **C**). All other hair cell nuclei are shown in blue. Inset figures show representative orthogonal optical sections of stained nuclei (grayscale). **(E–H′)**. Volume reconstructions of CALB2+ and biocytin-labeled dendrites illustrate the details of their projections in close proximity to hair cell nuclei. **(E,E′)** A 3D volume rendering of the corresponding boxed area in **A** illustrates bouton type endings (arrows) in close apposition to hair cell nuclei (magenta; **E′**). **(F,F′)** A 3D volume rendering of the corresponding boxed area in **A** shows a CALB2+ afferent wrapping around a hair cell nucleus (yellow) and a biocytin labeled neuron in close apposition to a hair cell nucleus (magenta). **(G,G′)** 3D volume renderings of the corresponding boxed region in **B** exhibiting a large-intermediate diameter biocytin labeled fiber forming a bowl at the base of a hair cell nucleus (cyan). **(H,H′)** 3D volume renderings of the corresponding boxed region in **B** show two CALB2+ fibers also labeled with TRITC-biocytin in close proximity to hair cells (nuclei shown in yellow). Note that CALB2 immunolabeling and TRITC-biocytin labeling closely overlap. Scale is the same for **(A–D)** (30 μm), and insets (72 μm). Scale is not shown for 3D volumes.)*

Although anti-TUBB3 immunohistochemistry revealed fine dendritic projections, it did not enable visualization of dendritic varicosities representing putative boutons of dimorphic afferent dendrites within the crista central zones or utricular striola (Fernandez et al., 1988, 1990). Therefore, retrograde labeling of TRITC-conjugated biocytin was used to visualize the fine dendritic branches and putative boutons innervating the central zones of superior and horizontal cristae and striolar region of the utricle. **Figures 11A,B** show TRITC-labeled fibers (represented in green) projecting to the horizontal canal crista and utricle 6 months after gentamicin treatment, illustrating patent fibers projecting to the central zone and striola of these epithelia. In addition, these fine dendritic branches have morphological features representative of *en passant* and terminal boutons, characterized by varicosities along the trajectories and at the terminations of thin dendritic branches (Fernandez et al., 1988, 1990). Extensive damage to this region is illustrated by the diminished hair cell densities observed in the maximum intensity projections of hair cell nuclei (**Figures 11C,D**). Three-dimensional reconstructions of representative labeled fibers further illustrate the varicosities on small diameter branches (arrows; **Figures 11E,F**) and show that large-intermediate diameter afferents with retracted calyces often were closely apposed to hair cell nuclei (**Figures 11G,H**). Some of these fibers exhibited a bowl-shaped “hemi-calyx” ending at the base of the closely apposed hair cell (**Figures 11G,G**′).

## Discussion

**FIGURE 12:**
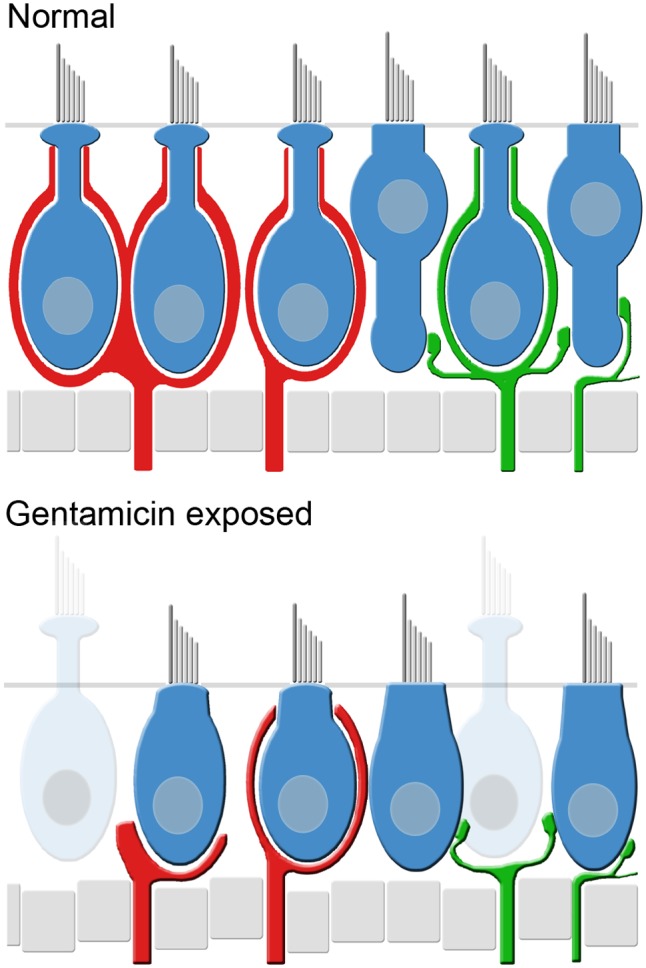
Summary schematic of partial lesion induced by low-dose gentamicin in the cristae central zones and utricle striola. The *Normal* (untreated) panel **(top)** depicts the components of these neuroepithelial regions, including types I and II hair cells exhibiting stereotyped morphologies, CALB2+ afferents with simple and complex calyces (i.e., calyx-only, red), dimorphic afferents (green), spherical hair cell nuclei, and tightly packed support cell nuclei. Vestibular epithelia exposed to *Low-dose gentamicin* **(bottom)**, however, undergo several alterations that characterize the lesion. These include: (a) retraction of most calyces, including those of dimorphic afferents (right side of panel; **Figures 3, 6, 7, 8**); (b) modest hair cell loss **(Figures 6, 9)**; (c) altered morphology of remaining hair cells, such that the stereotype morphologies of types I and II hair cells are not readily distinguished; these hair cells are viable as indicated by positive MYOVI immunolabeling **(Figure 10)**; (d) remaining non-CALB2+ afferents projecting to the crista central zones and utricular striola (i.e., afferents originally exhibiting dimorphic morphologies) retain fine dendritic branches and putative postsynaptic boutons **(Figure 11)**.

Through the present investigation we implemented a method for direct intraperilymphatic gentamicin administration to achieve three principal objectives. First, we sought to produce a limited lesion among hair cells of the vestibular epithelia, thereby unmasking potential effects on the dendrites of primary afferent neurons (i.e., to investigate dendritic lesions in cases of limited hair cell loss). Second, we believed the refined strategy would limit the dosing variability and, therefore, achieve greater consistency in the outcome parameters. Our third objective was to elucidate the effects of lesioning both potential targets (i.e., hair cells and afferents dendrites) on the discharge characteristics of afferent neurons. These results are summarized in the cartoon depicted in **Figure 12**, the discussion of which will include the limitations and impacts of the outcomes of this experimental approach.

### Limitations of the Present Results

#### Limited Hair Cell Loss Unmasks Independent Calyx Retraction

The administration of 1 μg gentamicin to the perilymph resulted in the loss of hair cells that was determined by comparing counts within the cristae central zones and utricular striolae from treated and contralateral control labyrinths. The mean hair cell counts ranged between 82.5 and 61.6% of hair cells in the contralateral labyrinths (0.5 and 6 month specimens, respectively), corresponding to mean hair cell loss estimates between 17.5 and 38.4%. Since the fraction of type I hair cells found in the central zones and striolae of untreated epithelia are approximately 60% (Desai et al., 2005a,b), the measured hair cell loss did not amount to the total number of type I hair cells. At the same time, virtually all calyces were lost in preparations investigated at 2 months post-administration and longer; **Figure 3** illustrates a case of extensive calyx loss even at 0.5 months. Some of the early calyx loss may be a downstream consequence of apoptosis (Forge and Li, 2000; Matsui et al., 2002, 2003, 2004; Cunningham et al., 2004; Ding et al., 2010; Zhang et al., 2012; Tao and Segil, 2015) among the most sensitive type I hair cells. However, the vast calyx loss found at the later post-administration times could not be wholly accounted for by loss of encapsulated hair cells. While the findings of the present study cannot exclude the possibility that calyx loss was induced by factors released by gentamicin-damaged (yet still viable) hair cells, these data also indicate a reasonable probability that gentamicin targeted the calyx directly. This component of the lesion did not involve loss of parent axons, which would have implied degeneration and perhaps apoptosis of Scarpa’s ganglion neurons (**Figure 8**). Therefore, the restricted lesion involving retraction of the calyx can be characterized as *non-apoptotic*. Data from the present study indicates that this lesion component may exhibit greater sensitivity, and perhaps have a greater impact, than hair cell *loss* in terms of the gentamicin-induced vestibular dysfunction or hypofunction.

While data of the present investigation do not provide direct insight into the pathophysiologic mechanisms of the lesions induced by low-dose gentamicin, they do provide a foundation upon which to build future investigations of the most labile components of vestibular dysfunction. In view of broad evidence indicating the deleterious action of gentamicin on mitochondria (Dehne et al., 2002; Morales et al., 2010), and the large number of mitochondria within afferent calyces (Lysakowski and Goldberg, 1997, 2008), it would be far from surprising if afferent calyces were directly susceptible to direct gentamicin-induced damage. The density of mitochondria within calyces implies that considerable energy production is required for normal homeostasis of this structure, for example, to maintain the activity of the robust expression of Na^+^/K^+^ATPases within calyceal membranes (Cheng et al., 2006; Schuth et al., 2014). Gentamicin-induced mitochondrial compromise may lead to deteriorating function of the membrane ATPases and the inability to maintain membrane potential within the calyx, eventually leading to its morphologic collapse and retraction. Again, this does not necessarily lead to afferent apoptosis, as the accounting of CALB2+ parent axons indicated their numbers were similar to that found in untreated labyrinths (**Figure 8**).

The present investigation did not enable resolution of the hair cell phenotypes that were lost upon gentamicin administration. The afferent calyx is the only morphologic characteristic that unambiguously identifies a type I hair cell. Other characteristics, such as general chalice morphology or nuclear location within the epithelia, are less definitive. Since gentamicin exposure, even at the low doses used in the present study, leads to calyx retraction and distortion of the epithelia that precludes the use of other characteristics (even if less reliable), it was not possible to estimate the contribution of hair cell phenotype to their total loss. Furthermore, the temporal profile of the loss suggests that there may be differential sensitivities whereby the most sensitive (amounting to approximately 18% within the crista central zones) are lost by 2 weeks, and another less sensitive group (amounting to an additional approximately 17%) that is lost over the succeeding 5.5 months. The proclivity with which type I hair cells have been shown to take up and retain gentamicin (Lyford-Pike et al., 2007) suggests that they constitute the most sensitive fraction to gentamicin. However, this group cannot constitute the full complement of type I hair cells, and other type I hair cells are likely to be as insensitive as type II hair cells (evidenced in **Figure 10H**).

#### Intraperilymphatic Administration and Outcome Variability

The goal of direct administration of gentamicin to the perilymph was to limit the variability in outcome measures to that associated with gentamicin pharmacology within vestibular epithelia and subsequent detection methods, eliminating variability associated with dosing and the quantity reaching the labyrinth. In only one preparation (0.5 months post-administration) was there no evidence of hair cell loss or calyx retraction, which we attribute to a failure of gentamicin administration. During port placement in this preparation, the subarcuate fossa adjacent to the bony superior canal was violated, which likely provided a pathway for misdirecting the infusate away from the labyrinth leading to the administration failure. Every other preparation resulted in hair cell loss and calyx pathology, and in preparations investigated at 1 month post-administration and longer severe attenuation of stimulus-evoked afferent discharge modulation was found. This supports the conclusion that these results provide a perspective of the inherent pathophysiologic variability of gentamicin administration that is independent of dosing variability. This includes: (1) hair cell and calyx loss that ensues within 2 weeks post-administration, exhibits modest variability through 1 month post-administration, but is highly stable after that time; (2) though severe response attenuation can be apparent within 2 weeks, some weak residual responsiveness can remain through 2 months; and (3) severe response attenuation persists through 6 months post-administration.

#### CALB2+ Calyces as an Index of Central Zone and Striolar Calyces

We used the subpopulation of CALB2+ calyces as the marker with which to delineate defined regions of vestibular epithelia [i.e., cristae central zones and utricular striolae (Desai et al., 2005a,b)] for hair cell counts, and as indices of calyceal morphology. While this approach certainly served its purpose, it did not specifically address whether, in specimens examined a 0.5, 1, and 2 months post-administration, all central zone calyces exhibited pathology that paralleled CALB2+ calyces (i.e., CALB2– calyces). We do provide evidence that CALB2+ calyx pathology was representative of all central zone and striolar calyces at 6 months post-administration (**Figures 6C,D**), including those beyond the central zone and striolar boundaries. CALB2+ calyx pathology that was not representative of other calyces at shorter post-administration intervals would be indicative of a differential sensitivity to gentamicin, for which we are unaware of any evidence. **Figures 6C,D** demonstrate the absence of any such differential sensitivity at 6 months post-administration. As noted above, hair cell loss could not be distinguished by phenotype, including those designated type I_c_ (those encapsulated by CALB2+ calyces) and I_d_ hair cells (those encapsulated by CALB2– calyces) (Li et al., 2008), providing no insight into this level of gentamicin sensitivity. While we expect that all central zone and striolar calyces exhibit similar pathology as represented by CALB2+ calyces, this remains an issue to be confirmed in future experiments.

#### Response Attenuation with Intact Spontaneous Discharge

Hirvonen et al. (2005) first reported the remarkable finding of severely attenuated stimulus-evoked discharge modulation and preserved spontaneous discharge in chinchillas that underwent intratympanic gentamicin administration. They provided evidence for average hair cell loss of 57% across crista specimens evaluated (from serial cryostat sections), and without reference to crista zone distinctions. In the present study, we focused specifically upon the crista central zones and utricular striolae based upon prevailing evidence for these regions being the most sensitive to aminoglycoside-induced pathology (Wersall and Hawkins, 1962; Watanuki et al., 1972; Lii et al., 2004; Lyford-Pike et al., 2007; Lue et al., 2009). The principal focus was to investigate afferent dendritic pathology that could be not be accounted for by regional hair cell loss. Therefore, utilizing more focused histologic analyses with more modest hair cell loss (18–34%), we estimate that the effective gentamicin dose in the present study was less than that responsible for the lesions induced in the previous study that produced greater hair cell loss (Hirvonen et al., 2005). Despite a gentamicin dose leading to less hair cell loss, our physiologic findings, including severely attenuated responses to natural stimuli and spontaneous discharge that exhibited greater mean ISIs than found in untreated afferents, resembled those previously reported (Hirvonen et al., 2005).

In the present investigation evoked discharge modulation in vestibular afferent neurons was evaluated using stimulus characteristics for which robust responses were readily detected in untreated specimens (**Figures 2, 4**). The detection of stimulus-mediated responses was evaluated using stimulus–response coherence measures, which were determined from comparisons of frequency spectra contained in raw spike trains (Jarvis and Mitra, 2001) and the applied stimuli. Each coherence measure was accompanied by a probability measure corresponding to chance correlation. The untreated afferents included in the figures were selected for their low response sensitivities, which presented the greatest challenge for the coherence analyses in demonstrating representative coherence measures among semicircular canal afferents. These examples at the designated stimulus characteristics exhibited typically high coherence measures, and very low probabilities of chance coherence between stimulus and response (**Figures 2, 4**). Most coherence measures determined from the discharge of gentamicin-treated labyrinths were low by comparison, and the probabilities of chance correlation were typically high. Of course, it is always possible that increasing stimulus magnitude (e.g., by increasing stimulus frequency and/or peak velocity) would result in higher coherence between stimulus and response, as demonstrated by the superior canal afferent in **Figures 4A-vii,viii**. This reflects a threshold elevation due to the gentamicin treatment, which is not normally observed in mammalian afferents at comparable stimulus characteristics. Therefore, we interpret the low coherence measures among semicircular canal afferents from gentamicin-treated specimens, or the absence of stimulus–response coherence, as a severe attenuation in the capabilities for stimulus-evoked discharge modulation.

A limitation of the present study concerns our inability to apply linear acceleration stimuli in the electrophysiologic evaluation of untreated or treated afferents. Hirvonen et al. (2005) reported that 45 of 212 afferents recorded from gentamicin-treated afferents exhibited severely attenuated responses, demonstrating that, even under morphologic lesions induced in their preparations, responses were detected in 21% of recorded afferents. This indicates the likelihood that a small fraction of afferents that exhibited low response coherence to rotational stimuli projected from the utricle. This fraction may be estimated to be approximately 4%, representing the estimated combined probability of 21% response probability from Hirvonen et al. (2005) and the 20% probability of encountering a utricular afferent from our electrophysiologic approach (see **Figure 2A**). However, it is also important to note that Hirvonen et al. (2005) also reported severe response attenuation was a feature of both canal and utricular afferents, providing evidence that the gentamicin-induced dysfunction was induced in both epithelia (Hirvonen et al., 2005). Consequently, application of linear acceleration stimuli in our preparations would have yielded low response coherence measures in an additional small fraction of afferents, and likely would not have had additional impact on the resulting conclusions. Therefore, our findings were similar in that modest responses were detected in a small fraction of afferents (e.g., **Figure 4**), but the absence of stimulus-evoked discharge was the predominant finding among most afferents recorded in the present study.

The preservation of spontaneous discharge among afferents recorded in the present study, in addition to remnant and attenuated stimulus-evoked discharge modulation, indicated that synaptic transmission between hair cell and afferent neurons remained intact, to some degree, following administration of low-dose gentamicin. **Figure 10H** illustrates that the anatomical substrate for connectivity exists between viable hair cells and remnant parent axons even after calyces have retracted in putative calyx-only (CALB2+) afferents (Fernandez et al., 1988; Desmadryl and Dechesne, 1992). It is more plausible that spontaneous and remnant evoked discharge was driven by synaptic transmission between type II hair cells and remaining postsynaptic afferent boutons that were shown to exhibit intact morphology (**Figure 11**). Type II hair cells have also been shown to project onto parent axons through lateral extensions near the support cell layer (Pujol et al., 2014). This projection provides the anatomical substrate for type II hair cells to drive spontaneous discharge in CALB2+ calyx-only afferents even under conditions in which the calyces have retracted.

If synaptic transmission between hair cells and afferent dendrites was intact following administration of low-dose gentamicin, what could be the mechanism responsible for the severe attenuation or elimination of stimulus-evoked discharge? It is certainly most likely that this result stems from an effect to hair cells that does not lead to apoptotic events. It was previously suggested that this result is consistent with compromise to the hair cell transduction apparatus (Hirvonen et al., 2005), possibly resulting from stereocilia fusion (Duvall and Wersall, 1964; Takumida et al., 1989a) or disruption of stereocilia tip links (Takumida et al., 1989b). Gentamicin-induced transduction channel blockade is a reversible effect subject to washout (Jaramillo and Hudspeth, 1991; Nagata et al., 2005; Alharazneh et al., 2011), and is unlikely to persist at the long post-administration times investigated in the present and previous study (Hirvonen et al., 2005). Alternatively, the coexistence of spontaneous discharge preservation and evoked discharge compromise is also consistent with the condition found in some vestibular hair cells in mice lacking the gene coding for the protein otoferlin. Dulon and colleagues (Dulon et al., 2009; Vincent et al., 2014) reported that striolar type I hair cells did not exhibit depolarization-induced increases in membrane capacitance, a proxy for stimulus-induced transmitter exocytosis, while spontaneous discharge was intact in such preparations. In type II hair cells, while the relationship between membrane capacitance and local calcium concentration was found to be altered, they did not exhibit the same dependence upon otoferlin as striolar type I hair cells (Dulon et al., 2009). Otoferlin immunolabeling was diminished in apical turn mouse inner hair cells at day 10 following daily systemic administration of gentamicin (ShuNa et al., 2009), suggesting that otoferlin may be labile to gentamicin treatment (e.g., through diminished expression or induction of a post-translation modification). These data suggest that gentamicin may induce a condition in hair cells that compromises effective stimulus-induced neurotransmitter release, while spontaneous discharge remains intact. This is unlikely to be the complete story in view of the intact function of type II hair cells, but the similarities in physiologic outcomes between otoferlin-null and gentamicin-treated preparations are compelling.

The data of the present investigation demonstrate that stimulus-evoked discharge modulation is severely attenuated after administration of intraperilymphatic low-dose gentamicin, despite the presence of viable hair cells. We have shown that this gentamicin dose resulted in more modest hair cell loss than previously shown (Hirvonen et al., 2005), and while direct evidence is not offered in the present study we presume this occurred primarily through apoptotic mechanisms (Forge and Li, 2000; Matsui et al., 2002, 2003; Cunningham et al., 2004; Zhang et al., 2012). In specimens that reflected modest hair cell loss, other effects not directly related to hair cell apoptosis dominated the morphologic and physiologic aspects of the lesion, and included complete calyx retraction and drastic attenuation in the capabilities for stimulus-evoked discharge modulation. This supports the conclusion that non-apoptotic effects overwhelm the apoptotic effects in the vestibular dysfunction that resulted from modest gentamicin exposure [present study, (Hirvonen et al., 2005)].

### Translational Impact on Clinical Applications of Intratympanic Gentamicin

#### Gentamicin-Induced Hypofunction

Intratympanic administration of gentamicin (ITG) has become a clinical treatment for conditions of intractable vertigo associated with Mèniére’s syndrome (Carey, 2004; Cohen-Kerem et al., 2004; Casani et al., 2014; Marques et al., 2015; Rah et al., 2015; Syed et al., 2015; Espinosa-Sanchez and Lopez-Escamez, 2016; Junet et al., 2016) and as pretreatment in advance of vestibular schwannoma resection (Magnusson et al., 2007, 2009). The goals of ITG treatments are to induce vestibular hypofunction that would provide a reduction in the occurrence of vertigo and expedite intrinsic central compensatory mechanisms, while preserving cochlear function (e.g., Carey, 2004; Huon et al., 2012; Marques et al., 2015; Espinosa-Sanchez and Lopez-Escamez, 2016). Evidence in support of the efficacy of this strategy is provided by Magnusson et al. (2007, 2009), who report that patients requiring vestibular schwannoma resection exhibit a drastically reduced incidence of vertigo when they undergo “prehab” treatments of gentamicin-induced hypofunction and physical therapy regimens to promote central vestibular compensation. Ostensibly, the preoperative hypofunction and subsequent central compensation dampen the effects of the abrupt unilateral vestibular ablation resulting from tumor resection. In general, the efficacy of ITG treatments can be variable, which most likely is due to dosing variability resulting from individual patient factors leading to heterogeneity in the quantity of gentamicin that diffuses into the perilymph. Crane et al. (2009) identified factors in individual patients that improved outcomes in a second ITG treatment following an initial unsuccessful application. Hence, under these conditions it is difficult to interpret whether variability in treatment efficacy is due to dosing or intrinsic heterogeneity in the cellular response to gentamicin.

While the present investigation utilizing normal animal subjects cannot provide direct insight into the etiology of Mèniére’s syndrome or other clinical conditions, it does provide important information regarding the pathophysiology of gentamicin-induced vestibular hypofunction. Direct intraperilymphatic administration eliminates potential dosing ambiguity associated with ITG, and while unlikely to be implemented as a clinical procedure it does provide insight into the outcomes that result under optimal administration conditions. Under the refined dosing conditions of the present investigation we found modest intrinsic variability within the first post-administration month across the limited number of subjects with respect to the morphologic (hair cell loss, calyx retraction) and physiologic (residual stimulus-evoked afferent discharge modulation) lesions. Hirvonen et al. (2005) reported similar findings, although the variability across subjects is difficult to assess in these data. In the present study, the induced lesion stabilized in the period between 2 and 6 months post-administration. Therefore, these findings provide insight into a temporal model for the development of hypofunction that may guide repeated treatment regimens.

A critical topic in ITG therapy for intractable Mèniére’s syndrome concerns the relative level of induced hypofunction that optimizes the reduction in vertigo. Recent reviews of the clinical literature concerning ITG therapy are available (Casani et al., 2014; Rah et al., 2015; Syed et al., 2015; Espinosa-Sanchez and Lopez-Escamez, 2016) and summarize the findings associated with residual function and vertigo control. The contribution of the present investigation, as well as that of Hirvonen et al. (2005), is in providing important perspectives regarding the pathophysiology associated with the hypofunction. As noted above, we estimate that the direct intraperilymphatic dose used in the present study [approximately 130 μM, based upon 1 μg in a perilymph volume of 16 μl (Shinomori et al., 2001)] was lower than that achieved by ITG in chinchillas (Hirvonen et al., 2005). This conclusion was based upon the relative magnitude of hair cell loss, which was approximately half in the present study. Both studies demonstrated severe stimulus-evoked response attenuation in afferent neurons, representing a non-apoptotic effect that was independent of hair cell loss. If the goal of ITG therapy is to induce hypofunction (i.e., and not complete ablation), then the outcomes demonstrated herein and by Hirvonen et al. (2005) would seem to represent the maximum desirable functional reduction. That is, spontaneous discharge among vestibular afferents is a functional attribute that would provide residual input to central vestibular circuitry that is likely to be beneficial for compensatory mechanisms. The pathophysiologic substrates of smaller lesions resulting from lower gentamicin doses have yet to be comprehensively explored. However, preliminary studies from our laboratory showed that smaller intraperilymphatic quantities of gentamicin (e.g., 0.5 and 0.75 μg) resulted in greater function among semicircular canal afferents, though deficits were still apparent (Hoffman et al., 2013).

The present study provides insight into the dose required to achieve the maximum level of hypofunction consistent with optimizing central compensation. That is, abrupt elimination of afferent input associated with vestibular schwannoma resection appears to be suboptimal in promoting central compensation, supporting the “prehab” strategy (Magnusson et al., 2007, 2009). We have shown that 1 μg administered to the chinchilla labyrinth [estimated at 16 μl, the measured volume of the guinea pig labyrinth (Shinomori et al., 2001)] preserves vestibular afferent spontaneous discharge while inducing severe attenuation in stimulus-evoked activity modulation. If this represents the maximum level of desired hypofunction, then this gentamicin concentration, or 130 μM, represents an initial estimate of the dose that would achieve a corresponding level of hypofunction for ITG therapy in humans. The volume of the human perilymphatic space is approximately 10 times that of the chinchilla [158.3 μl (Buckingham and Valvassori, 2001)]. Therefore, these results suggest that the gentamicin quantity estimated to achieve maximally desired level of vestibular hypofunction would be 10 μg.

#### Post-treatment Recovery of Function

It stands to reason that a critical goal of intraperilymphatic gentamicin is to provide permanent vertigo relief, and quite apart from the cases of treatment failure there is evidence that vertigo symptoms may return after initial relief following ITG treatment for Mèniére’s syndrome. De Waele et al. (2002) reported cases of patients that exhibited a return of a positive caloric response after 12 months, following an initial post-ITG period during which no responses were measured. The presence of caloric responses 12 months after ITG therapy, during which vestibular hypofunction was documented, is not inconsistent with the results of the present investigation. These authors assumed that the gentamicin-induced loss of vestibular function was due to hair cell loss, and therefore reconciled the recovery of caloric responses was due to hair cell regeneration within the horizontal cristae (De Waele et al., 2002). However, it is also likely that the induced loss was due to non-apoptotic functional deficits (e.g., loss of stimulus-exocytosis coupling) as demonstrated in the present results. This potential recovery of the non-apoptotic component of the gentamicin-induced lesion was found only after the 6 months examination [when vestibular dysfunction persisted in all patients (De Waele et al., 2002)]. The longest post-administration interval examined in the present study was 6 months, which the results of De Waele et al. (2002) would suggest is insufficient to observe any modest recovery of the non-apoptotic lesion. It may be possible that the recovery of caloric responses could have reflected a direct thermal effect upon spontaneously discharging afferents (Peterka et al., 2004). However, the recovery was also accompanied by the absence of refixation saccades after head impulse testing, which would only result under conditions of functional hair cells and vestibulo-ocular reflex (De Waele et al., 2002). The absence of functional recovery following gentamicin-induced hypofunction at post-administration periods ≤6 months was also indicated the results by Hirvonen et al. (2005) and Bremer et al. (2014). The results of De Waele et al. (2002) are intriguing, and may be indicative of long-term recovery capabilities for non-apoptotic lesions.

### Summary

While the use of small gentamicin doses unmasked a potentially critical effect on vestibular primary afferent dendrites, it also provides insight into other important aspects of vestibular pathophysiology. Certainly, establishment of precise ototoxicity protocols that render partial lesions to the vestibular epithelia are invaluable to the development of testing strategies for the early diagnosis of peripheral vestibular dysfunction. It also establishes a platform for the development of strategies for protection against ototoxicity resulting from systemic aminoglycoside therapy or chemotherapy. Furthermore, it must be recognized that the intratympanic administration (ITG) of gentamicin for the treatment of Mèniére’s disease is being conducted without an animal model exhibiting comparable level of pathology or dysfunction. That is, while partial dysfunction has been demonstrated in patients receiving ITG (Nguyen et al., 2009), a pathophysiologic model exhibiting similar hypofunction has not been produced. Such a model would be extremely valuable in providing a comprehensive pathophysiologic picture of the induced pathology.

## Author Contributions

DS and LH made substantial, direct, and intellectual contributions to the conception, execution, and analyses of the experimental work described, and approved it for publication.

## Conflict of Interest Statement

The authors declare that the research was conducted in the absence of any commercial or financial relationships that could be construed as a potential conflict of interest.
